# Possible osteosarcopenia is associated with loss in intrinsic capacity and early cognitive deficits: a longitudinal study of older adults in Singapore

**DOI:** 10.1093/gerona/glag211

**Published:** 2026-08-26

**Authors:** Vanessa J W Koh, T M T Khong, Lakkhina Troeung, Rahul Malhotra, Dheeraj Jha, Anitha D Praveen, Y X Liu, Benedikt Helgason, Navrag B Singh, Angelique W M Chan

**Affiliations:** Program in Health Services and Systems Research & Population Health, Duke-NUS Medical School, Singapore, Singapore; Centre for Ageing Research & Education (CARE), Duke-NUS Medical School, Singapore, Singapore; Centre for Ageing Research & Education (CARE), Duke-NUS Medical School, Singapore, Singapore; Centre for Ageing Research & Education (CARE), Duke-NUS Medical School, Singapore, Singapore; Program in Health Services and Systems Research & Population Health, Duke-NUS Medical School, Singapore, Singapore; Centre for Ageing Research & Education (CARE), Duke-NUS Medical School, Singapore, Singapore; Future Health Technologies, Singapore-ETH Centre, Campus for Research Excellence and Technological Enterprise (CREATE), Singapore, Singapore; Future Health Technologies, Singapore-ETH Centre, Campus for Research Excellence and Technological Enterprise (CREATE), Singapore, Singapore; Future Health Technologies, Singapore-ETH Centre, Campus for Research Excellence and Technological Enterprise (CREATE), Singapore, Singapore; Future Health Technologies, Singapore-ETH Centre, Campus for Research Excellence and Technological Enterprise (CREATE), Singapore, Singapore; Institute for Biomechanics, ETH Zürich, Zürich, Switzerland; Future Health Technologies, Singapore-ETH Centre, Campus for Research Excellence and Technological Enterprise (CREATE), Singapore, Singapore; Program in Health Services and Systems Research & Population Health, Duke-NUS Medical School, Singapore, Singapore; Centre for Ageing Research & Education (CARE), Duke-NUS Medical School, Singapore, Singapore; (Medical Sciences Section)

**Keywords:** Osteoporosis, Sarcopenia, Intrinsic capacity, Episodic memory, Healthy aging

## Abstract

**Background:**

Osteosarcopenia is linked to frailty, falls, fractures, and mortality. It may impair intrinsic capacity (IC), with emerging evidence especially pointing to its relationship with cognition capacity. We determined the prevalence and characteristics of persons with possible osteosarcopenia in older adults and examined its association with IC, with a focus on cognition.

**Methods:**

Two thousand, two hundred ninety-one participants were recruited through a national household survey of older adults in Singapore. Socio-demographic, health, and psychosocial variables were collected using questionnaires and physical assessments at baseline. Possible osteosarcopenia was defined by the concurrent presence of possible osteoporosis and possible sarcopenia. Multilevel mixed‑effects regression models examined the association of possible osteosarcopenia with IC at baseline and with cognitive function at 6 months post-baseline.

**Results:**

Possible osteosarcopenia, observed among 50.3% of the cohort, was associated with lower IC at baseline, including cognition capacity. Furthermore, baseline possible osteosarcopenia was associated with poor cognitive function at 6 months post-baseline, particularly among those with loss in cognitive capacity at baseline. This was driven by impaired delayed recall and poor orientation abilities, indicators of both early onset and gradual decline of cognitive impairment. On the other hand, higher education, social networks, resilience, and quality of life were associated with better IC (at baseline) and cognition (at baseline and at 6 months post-baseline).

**Conclusions:**

Possible osteosarcopenia is prevalent among older adults in Singapore and is associated with loss in intrinsic capacity and cognitive function, highlighting the importance of early intervention in rapidly aging societies.

## Introduction

By 2050, the proportion of adults aged 65 years and older is estimated to reach 16% globally.^1^ In Singapore, the setting of this study, 25% of the resident population is projected to be 65 years and older by 2030,^2^ making it one of the fastest aging countries in the world. Healthy aging depends on the ability to sustain the reserves of multiple physiological systems.^3^ The musculoskeletal system, a metabolic reservoir and central to one’s mobility and independence, experiences age-related decline over time,^4^ elevating the risk of osteoporosis and sarcopenia among older adults. Osteoporosis is a chronic bone metabolic disease characterized by low bone mineral density (BMD) and impaired bone mass and microstructure.^5^ Sarcopenia, a skeletal muscle disease characterized by decline in muscle mass, strength, and/or physical function,^6^ often co-exists with osteoporosis. The term “osteosarcopenia” has been proposed to define the concurrent presentation of osteoporosis and sarcopenia.

A meta-analysis of 66 studies indicated that the global pooled prevalence of osteosarcopenia was 18.5%, with Asia having a slightly higher prevalence compared to other regions (21.6%).^7^ Some studies have demonstrated the increased risk of frailty, falls, and fractures among those with osteosarcopenia.^8^ The adverse consequences of injurious falls and fractures not only lead to physical and psychological damage among individuals but also result in large healthcare and economic pressures. A recent study in Singapore projected that the incidence of osteoporotic fractures is projected to increase by 60%, growing to SGD $289.6 million by 2035.^9^

However, healthy aging extends beyond physical health and frailty. The World Health Organization (WHO) released a global report on aging and health in 2015, introducing the concept of intrinsic capacity (IC) as a framework to understanding the multidimensional nature of aging. IC, defined as “*the composite of all the physical and mental capacities of an individual*,” is conceptualized as 5 capacities: locomotion, cognition, psychological, vitality, and sensory.^10^ This framework offers a unique lens to examining osteosarcopenia and its potential impacts on aging.

While limited studies have investigated the association between osteosarcopenia and IC, some evidence suggests that the significance of osteosarcopenia may extend beyond the musculoskeletal consequences. Recently, a US cohort study reported an association between osteosarcopenia and poor performance in selected cognitive domains, particularly processing speed and memory, which was primarily driven by impaired muscle strength.^11^ A cross-sectional association between osteosarcopenia and cognitive impairment suggested that the deterioration of musculoskeletal reserves may be an indicator of broader physiological decline.^12^ Moreover, a dose-response relationship between cognition and mortality have been observed, where older adults with cognitive impairment had increasing mortality risk with increasing severity of impairment.^13^ Cognitive impairment contributes significantly to overall healthcare costs as well, estimating at USD $124,000 per capital lifetime burden and totaling to USD $630 billion in aggregate.^14^ Hence, understanding the relationship between osteosarcopenia and cognitive function could be valuable in providing insights for early identification and preventive strategies for cognitive decline.

Emerging evidence suggests that osteosarcopenia may be a key contributing factor in both cognitive and physical health in older adults. Yet its epidemiology and implications on health and intrinsic capacity among older adults remain insufficiently characterized globally. Its impact on multidimensional intrinsic capacity remains poorly characterized, with existing studies limited to cross-sectional designs and separate studies on osteoporosis and/or sarcopenia only.^15^^,^^16^ Evidence from Asian populations on the relationship between osteosarcopenia and intrinsic capacity is also scarce, despite well-documented differences in body composition and muscle mass thresholds among Asians.^17^ Additionally, the limited evidence extends studies conducted on cognition as well when comparing studies conducted in Asia versus the West.^18^^,^^19^ This knowledge gap is particularly critical given that geriatric syndromes such as cognitive impairment, falls, osteoporotic and fragility fractures^20–22^ result in significant direct burden on individuals and healthcare systems. Using Singapore as a model for aging countries, especially in the Asian context, this study characterized the epidemiology and health implications of osteosarcopenia. Osteosarcopenia was assessed using validated questionnaires and anthropometric assessments,^23^^,^^24^ where individuals meeting both osteoporosis and sarcopenia criteria were classified as having *possible osteosarcopenia* (details in Methods).

The primary aim of this study was to assess the association between baseline possible osteosarcopenia and IC and the effect of baseline osteosarcopenia on cognitive function at 6 months post-baseline. The secondary aim was to estimate the prevalence and describe the characteristics of individuals with possible osteosarcopenia in Singapore. Findings from this study provide evidence for osteosarcopenia in similar aging populations facing comparable healthcare challenges.

## Methods

### Study design

Data were analyzed from the Targeted Assessment and Recruitment of Geriatrics for Effective fall prevention Treatments (TARGET) study, an ongoing national prospective cohort study of 2291 community-dwelling Singapore residents aged ≥60 years.^25^ Ethics approval was granted by the National University of Singapore Institutional Review Board (NUS-IRB-2021-168). All aspects of study reporting conformed to the STROBE guidelines (Table S1).

### Data collection

At baseline, participants completed a 2-hour home-based assessment conducted by trained interviewers, comprising (1) questionnaires on sociodemographic, environmental, clinical, functional, cognitive, and psychosocial status, and (2) a 5-minute walk test to assess gait using wearable sensors (ZurichMOVE).^26^ Those without a history of bilateral hip replacement were invited for a clinic visit for a dual energy X-ray absorptiometry (DXA) scan to assess BMD on a voluntary basis, resulting in 895/2291 (39.1%) participants undergoing the scans. Following baseline (*T*_0_), participants are followed up via telephone every 3 months for 2 years (*T*_1_ − *T*_8_) to prospectively monitor falls, fractures, and cognitive status.

## Measures

### Osteosarcopenia

#### Possible osteoporosis

Risk of osteoporosis was assessed using the Osteoporosis Self-Assessment Tool for Asians (OSTA).^23^^,^^27^ The OSTA index was calculated as:


0.2×[Body weight (kg)-Age (years)].

Based on the index, participants were categorized into three risk groups: high (OSTA < –4), moderate (OSTA –4 to –1), and low (OSTA > –1). Consistent with epidemiological studies in Asian populations, individuals in the moderate- and high-risk categories were grouped and classified as having *possible osteoporosis* while those in the low-risk category were classified as *no possible osteoporosis.*^23^^,^^28^ This classification reflects a screening-based risk stratification approach rather than a clinical diagnosis, as OSTA is designed to identify individuals at increased risk of low bone mineral density. Prior studies have defined individuals with intermediate and high OSTA scores as “at risk” and demonstrated that using a threshold of ≤ –1 improves sensitivity for osteoporosis in population-based settings.^28^^,^^29^

#### Possible sarcopenia

Based on the 2019 Asian Working Group for Sarcopenia (AWGS19) guidelines, possible sarcopenia was defined by low muscle strength assessed using handgrip strength.^24^ Handgrip strength was measured twice in each hand with a Smedley spring-type dynamometer (Tanita 6103, Tokyo, Japan) and recorded to the nearest 0.5 kg.^30^ The mean of the two measurements from the dominant hand was used. *Possible sarcopenia* was defined as handgrip strength <18 kg for women and <28 kg for men.^24^ Individuals with handgrip strength above these thresholds were classified as *no possible sarcopenia*. The 5-time chair stand test was not administered in TARGET; therefore, this component of the AWGS 2019 operational definition could not be included.

#### Possible osteosarcopenia

Individuals meeting the criteria for both possible sarcopenia and possible osteoporosis were classified as having *possible osteosarcopenia*. All others were classified as *no possible osteosarcopenia*.

We compared the possible osteosarcopenia classification based on the OSTA index and AWGS19-defined handgrip strength cutoff against the current gold standard diagnostic criteria for osteosarcopenia using DXA-derived BMD, muscle mass, and muscle strength (Table S2) among 895 participants who underwent DXA scans. Classification performance was evaluated through receiving operating curve (ROC) characteristics. The sensitivity of the possible osteosarcopenia classification was 92.3% (95% CI: 85.9% to 96.4%), specificity was 71.4% (95% CI: 66.6% to 75.8%) with a ROC performance of 82.0% (95% CI: 79.0% to 85.0%; Table S3), demonstrating its effectiveness as a proxy for classifying osteosarcopenia status. Nonetheless, it is important to note that *possible osteosarcopenia* is utilized as a proxy classification for the purposes of screening and should not be considered equivalent to clinically diagnosed osteosarcopenia.

### Intrinsic capacity

In line with the WHO definition, IC was assessed across 5 capacities: cognition, psychological, vitality, locomotion, and sensory function (vision and hearing; Table 1)^10^. While some studies have used exploratory or confirmatory factor analysis to reveal subfactors of IC,^31^ others have determined IC status by assigning a score to each of the 5 constituent domains—score of 0 (loss) or 1 (intact)^32^—before summing the domain scores. To align with global literature and ease of classifying IC in clinical settings, we derived a total IC score using the latter approach.

**Table 1:** Criteria for 5 domains of intrinsic capacity.

Domain	Component	Intrinsic capacity scoring (0-5)	
		0 (Loss in IC)	1 (Intact IC)
Cognition	MoCA score	≤25	≥26
Psychological	PHQ-9 score	≤5	≥6
Vitality	Body mass index	<18.5 kg/m^2^	≥18.5 kg/m^2^
Locomotion	Gait speed	≤0.8 m/s	>0.8 m/s
Sensory	Self-reported vision and hearing status	Self-reported vision or hearing rated as “Fair” or “Poor”	Self-reported vision and hearing rated as “Excellent” or “Very good” or “Good”

Abbreviations: MoCA, Montreal Cognitive Assessment; PHQ-9, Patient Health Questionnaire.

*Cognitive capacity* was assessed using the Montreal Cognitive Assessment (MoCA; range: 0 to 30), which evaluates episodic memory (delayed recall), visuospatial abilities (cube drawing, clock drawing), executive function (trail making test, verbal fluency, and abstraction). attention, concentration, working memory (cancellation, subtraction, digit span), language (naming, sentence repetition), and orientation. Adjusted MoCA scores were computed by adding one point to individuals with ≤12 years of formal education. Participants scoring ≤25 were classified as having impaired cognition (loss in cognitive capacity), while those scoring ≥26 were classified as having intact cognition (intact cognitive capacity).^33^

*Psychological capacity* was assessed using the Patient Health Questionnaire (PHQ-9; total score range: 0 to 27).^34^ Participants were asked how often, over the past 2 weeks, had they been bothered by depressive symptoms such as depressed mood, anhedonia, hopelessness, fatigue, loss of appetite, difficulty concentrating, or suicidal thoughts. Scores of 6–27 were classified as clinical major depression (loss in psychological capacity), while scores of 0–5 were considered intact psychological capacity.^35^

*Vitality capacity* was assessed with using body mass index (BMI) with cutoffs adapted from the Malnutrition Universal Screening Tool. Nutritional risk was indicated with a BMI <18.5 kg/m^2^ (loss in vitality capacity), while a BMI ≥18.5 kg/m^2^ was considered no nutritional risk (intact vitality capacity).^36^^,^^37^

*Locomotion capacity* was assessed by gait speed during a 5-min walk test under field-settings, for instance, in participants’ home or in corridors outside their home, using ZurichMove wearable sensors. Each participant was instructed to walk naturally at their comfortable speed. Linear acceleration and angular velocity signals from inertial measurement unit (IMU) sensors were recorded during the walk test and were filtered and analyzed with a validated gait event detection algorithm.^26^^,^^38^ Detected gait events were then used to identify key phases in all the gait cycles over the 5 min. Spatio-temporal parameters, for instance, stride time and length, cadence, and double-limb support time, and inter-cycle variations were calculated during the 5-min walk test to capture fluctuations and variability in movement patterns.^39^ The standard clinical cutoff of 1.0 m/s, as recommended by the AWGS 2019 guidelines, was not applied in this study.^24^ Instead, a lower threshold of 0.8 m/s was adopted to account for the nonclinical assessment environment, as gait measurements were conducted in participants’ homes rather than in controlled clinical settings.^40^ Participants with gait speed <0.8 m/s were classified as having loss in locomotion capacity, while those with gait speed ≥0.8 m/s were considered as having intact locomotion capacity).^24^

*Sensory capacity* was assessed through self-reported vision and hearing status. Sensory capacities were considered intact if participants reported “Excellent,” “Very good,” or “Good” for both items (intact sensory capacity). Those reporting “Fair” or “Poor” for either item were classified as loss in sensory capacity.^41^

### Cognitive function at 6 months post-baseline

This was assessed using the abbreviated version of the MoCA (mini-MoCA),^42^ which provides a continuous score (range: 0 to 15), where higher scores indicate better cognitive function. It includes 3 domains: language fluency, orientation, and delayed recall.

At 6 months post-baseline, 97.4% (2231/2291) participants were successfully followed up. Of these, 1228 (55.0%) completed all 3 mini-MoCA domains. Comparing baseline characteristics between those who did and did not complete the mini-MoCA, the latter were older, had lower MoCA scores, less physically active, frailer and more dependent on others for daily activities (Table S4). The sample sizes varied slightly for the individual domains—language fluency (*n* = 1248), orientation (*n* = 1234), and delayed recall (*n* = 1228)—as some participants did not complete all components.

### Covariates

We collected baseline data on a range of potential factors associated with sarcopenia and osteoporosis (Table S5). These included sociodemographic, health-related, lifestyle, and psychosocial variables.

### Statistical analysis

All analyses were conducted using Stata 18 (StataCorp LLC, College Station, TX). Participants were classified into two groups: *possible osteosarcopenia* and *no possible osteosarcopenia*. Descriptive statistics were used to compare baseline characteristics between the two groups. Student’s *t*-tests were applied for continuous variables, while chi-square tests were used for categorical variables.

Given the high proportion of missing mini-MoCA scores at 6-month follow-up (1063/2291, 45%), multiple imputation by chained equations (MICE) was applied to impute missing cognitive outcome scores. MICE does not assume a joint multivariate normal distribution but instead employs separate conditional distributions for each variable with missing data, making it suitable for our model.^43^^,^^44^ For total mini-MoCA score and individual domain scores (language fluency, orientation, and delayed recall), we imputed missing outcome scores using the same set of predictors (including interaction terms), specified in the corresponding nonimputed analysis. The main analyses presented below were performed on the 50 imputed data sets. It is also important to note that both osteoporosis and sarcopenia alone were not significantly associated with baseline intrinsic capacity or 6-month cognitive function; hence, we only reported the results for osteosarcopenia in the manuscript.

To examine the association of possible osteosarcopenia with IC at baseline and with cognitive function at follow-up, multilevel mixed-effects regression models with robust standard errors were employed to account for clustering within households. An unconditional null multi-level model was first run to estimate the intraclass correlation coefficient (ICC), which indicated how much total variability in cognitive function at 6 months post-baseline was attributed to cluster-level differences. The ICC (0.322) indicated that cognitive function of participants residing in the same household was highly correlated, necessitating the use of multilevel models. We also tested for potential effect modification by baseline cognitive capacity (i.e., 0: Loss in capacity/1: Intact capacity) in all longitudinal models to determine if the effect of possible osteosarcopenia on cognitive function at 6 months post-baseline was conditional on baseline cognitive capacity. To do this, the main effects for baseline possible osteosarcopenia and cognitive capacity, together with their interaction term, were included in all longitudinal models.

Covariates were selected through backward elimination, where variables with *p *>0.05 were excluded. Backward elimination is commonly used to improve the efficiency of multivariable modeling estimations.^45^ While this approach may limit comparability across models, it was chosen to increase accuracy, avoid overfitting, and retain variables that showed meaningful associations with each outcome. Results from the multilevel mixed-effects regression models were presented as odds ratios and regression coefficients with 95% confidence intervals. A two-tailed *p*-value <0.05 was considered statistically significant.

### Sensitivity analysis

Given the high proportion of missing 6-month mini-MoCA scores, we conducted a sensitivity analysis to test the robustness of our main analyses, which excluded participants with imputed values (*n* = 1228), and conducted the same regression models as above.

## Results

### Baseline characteristics and group differences by possible osteosarcopenia status

A total of 2291 participants completed the TARGET study baseline. The mean (standard deviation) age was 74.6 years (7.9), 57.9% were female, 66.4% were of Chinese ethnicity, and 65.3% had attained primary or secondary education. The majority reported adequate financial resources (75.6%), lived in public housing with 3 to 4 rooms (60.5%), and resided with family members or others (89.4%).

The prevalence of possible osteosarcopenia in the study participants at baseline was 50.3%. Compared to those without possible osteosarcopenia, participants with possible osteosarcopenia were significantly more likely to be older, of Chinese ethnicity, have had less formal education, reported occasionally adequate or usually inadequate financial resources, have had limited social networks, had have ≥4 chronic conditions, had have greater dependence in instrumental activities of daily living (IADLs), have had lower quality of life, used more medications, were be more frailer, and reported greater fear of falling, loneliness scores, and insomnia symptoms (Table 2 and Table S6).

**Table 2:** Selected characteristics of the study participants at baseline (*n* = 2291).

Characteristic	Total (*N* = 2291)^a^	No possible osteosarcopenia (*N* = 1127; 49.2%)	Possible osteosarcopenia (*N* = 1153; 50.3%)	*p*-Value
Age (*Mean* [*SD*])	74.6 (7.9)	70.4 (6.6)	78.6 (6.8)	<.001
**Age (*N*, %)**				<.001
60-64	302 (13.2)	270 (24.0)	31 (2.7)	
65-69	388 (16.9)	294 (26.1)	93 (8.1)	
70-74	362 (15.8)	209 (18.5)	151 (13.1)	
75-79	614 (26.8)	243 (21.6)	366 (31.7)	
80-84	380 (16.6)	97 (8.6)	282 (24.5)	
85+	245 (10.7)	14 (1.2)	230 (20.0)	
**Gender (*N*, %)**				.971
Male	964 (42.1)	473 (42.0)	486 (42.2)	
Female	1327 (57.9)	654 (58.0)	667 (57.9)	
**Ethnicity (*N*, %)**				.005
Chinese	1521 (66.4)	707 (62.7)	807 (70.0)	
Malay	433 (18.9)	246 (21.8)	185 (16.1)	
Indian	290 (12.7)	152 (13.5)	137 (11.9)	
Other	47 (2.1)	22 (2.0)	24 (2.1)	
**Highest educational level (*N*, %)**				<.001
No formal education	367 (16.0)	90 (8.0)	275 (23.9)	
Primary	741 (32.3)	327 (29.0)	409 (35.5)	
Secondary	757 (33.0)	423 (37.5)	332 (28.8)	
Vocational or Institute of Technical Education (ITE)	44 (1.9)	32 (2.8)	11 (1.0)	
Junior college (JC) or General Certificate of Education (GCE) “A” level	138 (6.0)	89 (7.9)	49 (4.3)	
Polytechnic	93 (4.1)	72 (6.4)	21 (1.8)	
University and above	151 (6.6)	94 (8.3)	56 (4.9)	
**Financial resources (*N*, %)^b^**				.029
More than adequate	72 (3.1)	38 (3.4)	34 (3.0)	
Adequate	1731 (75.6)	877 (77.8)	845 (73.3)	
Occasionally adequate	400 (17.5)	163 (14.5)	236 (20.5)	
Usually inadequate	83 (3.6)	46 (4.1)	36 (3.1)	
**Housing categories (*N*, %)**				<.001
HDB flat (1-2 rooms)	258 (11.3)	113 (10.0)	145 (12.6)	
HDB flat (3-4 rooms)	1387 (60.5)	650 (57.7)	731 (63.4)	
HDB flat (5 rooms and above) and private housing/others	646 (28.2)	364 (32.3)	277 (24.0)	
**Living arrangements (*N*, %)**				
Living alone	243 (10.6)	112 (9.9)	130 (11.3)	<.001
Living with child and spouse	699 (30.5)	420 (37.3)	274 (23.8)	
Living with child only	350 (15.3)	121 (10.7)	228 (19.8)	
Living with spouse only	719 (31.4)	340 (30.2)	376 (32.6)	
Living with others only (include foreign domestic worker)	280 (12.2)	134 (11.9)	145 (12.6)	
Intrinsic capacity score (*M* [*SD*])	2.8 (1.0)	3.1 (0.9)	2.4 (1.0)	<.001
**Locomotion domain (*N*, %)^b^**				<.001
0: Loss in capacity	1903 (83.1)	903 (80.1)	993 (86.1)	
1: Intact capacity	248 (10.8)	168 (14.9)	80 (6.9)	
**Vitality domain (*N*, %)^b^**				<.001
0: Loss in capacity	139 (6.1)	31 (2.8)	108 (9.4)	
1: Intact capacity	2148 (93.8)	1095 (97.2)	1045 (90.6)	
**Cognition domain (*N*, %)^b^**				<.001
0: Loss in capacity	1364 (59.5)	482 (42.8)	874 (75.8)	
1: Intact capacity	926 (40.4)	645 (57.2)	278 (24.1)	
**Psychological domain (*N*, %)^b^**				<.001
0: Loss in capacity	176 (7.7)	61 (5.4)	112 (9.7)	
1: Intact capacity	2111 (92.1)	1066 (94.6)	1037 (89.9)	
**Sensory domain (*N*, %)**				<.001
0: Loss in capacity	597 (26.1)	214 (19.0)	380 (33.0)	
1: Intact capacity	1694 (73.9)	913 (81.0)	773 (67.0)	
Appendicular lean mass (*M* [*SD*])^b^	16.1 (3.9)	17.2 (4.1)	14.4 (2.9)	<.001
**Sarcopenia (*N*, %)^b^**				<.001
No possible sarcopenia	854 (37.0)	852 (75.6)	0 (0.0)	
Possible sarcopenia	1429 (61.9)	275 (24.4)	1153 (100.0)	
**Osteoporosis (*N*, %)^b^**				<.001
No possible osteoporosis	648 (28.1)	647 (57.4)	0 (0.00)	
Possible osteoporosis	1640 (71.1)	480 (42.6)	1153 (100.0)	

Abbreviation: HDB, Housing & Development Board; GCE, General Certificate of Education.

a Osteosarcopenia status could not be determined for 11 participants, hence total number does not add up to 2291.

b Percentages do not add up to 100% due to missing values.

### Association between possible osteosarcopenia and IC

On average, participants had an IC score of 2.8 out of 5. Locomotion capacity was the most affected (intact capacity: 10.8%), followed by cognition (intact: 40.4%), sensory (intact: 73.9%), psychological (intact: 92.1%), and vitality (intact: 93.8%). Participants with possible osteosarcopenia had poorer overall IC scores and fewer intact individual IC domains compared to those without possible osteosarcopenia (Table 2).

Participants with possible osteosarcopenia had significantly lower IC scores compared with those without possible osteosarcopenia (*β* = –0.21, 95% CI: –0.29 to –0.14; *p* < .01) after adjusting for covariates (Table 3). Other variables linked to reduced IC included older age, limited financial resources, ≥4 chronic conditions, greater fear of falling, and higher loneliness scores. Secondary school education and above, better quality of life, and greater social networks were positively associated with greater IC scores. Moreover, those with possible osteosarcopenia at baseline had lower odds of intact capacity in all domains. However, this was statistically significant only in the vitality and cognitive domains (Tables S10 and S11).

**Table 3:** Association between possible osteosarcopenia and intrinsic capacity score at baseline (*n* = 2280).^a^

	Intrinsic capacity score		
	Coefficient	95% CI	*p*-Value
**Osteosarcopenia (Ref: No possible osteosarcopenia)**			
Possible osteosarcopenia	−0.21	(−0.29, −0.14)	<.001
Age	−0.02	(−0.03, −0.02)	<.001
**Highest educational level (Ref: No formal education)**			
Primary	0.05	(−0.05, 0.14)	.360
Secondary	0.27	(0.16, 0.37)	<.001
Vocational or Institute of Technical Education (ITE)	0.22	(−0.02, 0.46)	.073
Junior college (JC) or GCE “A” level	0.57	(0.41, 0.72)	<.001
Polytechnic	0.52	(0.34, 0.70)	<.001
University and above	0.56	(0.40, 0.71)	<.001
**Financial resources (Ref: More than adequate)**			
Adequate	−0.02	(−0.21, 0.16)	.812
Occasionally adequate	−0.20	(−0.40, 0.00)	.048
Usually inadequate	−0.34	(−0.59, −0.09)	.008
**Number of chronic conditions (Ref: No)**			
1-3 chronic conditions	−0.01	(−0.11, 0.08)	.814
≥4 chronic conditions	−0.12	(−0.23, −0.01)	.039
EuroQol-5 Dimension (EQ-5D) Quality of life	0.89	(0.61, 1.18)	<.001
Lubben Social Network Scale	0.01	(0.00, 0.02)	<.001
Loneliness score	−0.07	(−0.10, −0.04)	<.001
Iconographical Falls Efficacy Scale (ICON-FES) (fear of falling)	−0.02	(−0.02, −0.01)	<.001

a Osteosarcopenia status could not be determined for 11 participants, who were excluded for this analysis.

### Association between possible osteosarcopenia and cognitive domain of IC

Three in four (75.8%) participants with possible osteosarcopenia had loss in cognitive capacity, while this proportion was lower (42.8%) among those without possible osteosarcopenia (Table 2). In the regression model, compared to participants without possible osteosarcopenia, those with possible osteosarcopenia had significantly lower odds of intact cognitive capacity at baseline, with a 55% reduction (OR = 0.45, 95% CI: 0.34 to 0.60; *p* < .001) after adjusting for covariates (Table S11). Older age was also associated with lower odds of intact cognitive capacity, with each additional year linked to an 12% reduction in odds. Participants of Malay and Indian ethnicities had reduced odds of intact cognitive capacity compared to Chinese, by 66% and 50%, respectively. Limited financial resources and greater dependency in IADLs were also associated with lower odds of having intact cognitive capacity. Greater social networks were positively associated with intact cognitive capacity.

### Association between possible osteosarcopenia and cognitive function at 6 months post-baseline

At 6 months post-baseline, the mean mini-MoCA score among participants with baseline possible osteosarcopenia was 10.0 (range 0-15), while among participants without baseline osteosarcopenia it was 12.2. These scores indicate early to mild cognitive impairment.

The interaction term between possible osteosarcopenia and cognitive capacity at baseline was statistically significant, indicating that the effect of possible osteosarcopenia on cognitive function at 6 months post-baseline was conditional on the individual’s baseline cognitive capacity. Among those with loss in cognitive capacity at baseline, possible osteosarcopenia was associated with an additional 0.59-unit reduction in mini-MoCA scores at 6 months post-baseline (Figure 1 and Table 4). When examining those with intact cognitive capacity at baseline, there was no statistically significant difference in cognitive scores at 6 months post-baseline between those with or without possible osteosarcopenia (Table S12). Overall, compared to those without possible osteosarcopenia and with intact cognitive capacity at baseline, those with possible osteosarcopenia and loss in cognitive capacity at baseline is associated with an additional 1.74-unit reduction (over a score of 15) in mini-MoCA scores. It is important to note that the 1.74-unit reduction reflects the joint additive contrast between individuals with both possible osteosarcopenia and baseline cognitive loss relative to those with neither exposure, as estimated under the interaction model. This does not represent the isolated effect of possible osteosarcopenia alone of −0.59 units. Other factors associated with poorer Mini-MoCA score at 6 months post-baseline included older age, limited financial resources, and frailty at baseline. Interestingly, we also observed a positive association between the ≥4 chronic conditions at baseline and cognitive function.

**Figure 1:**
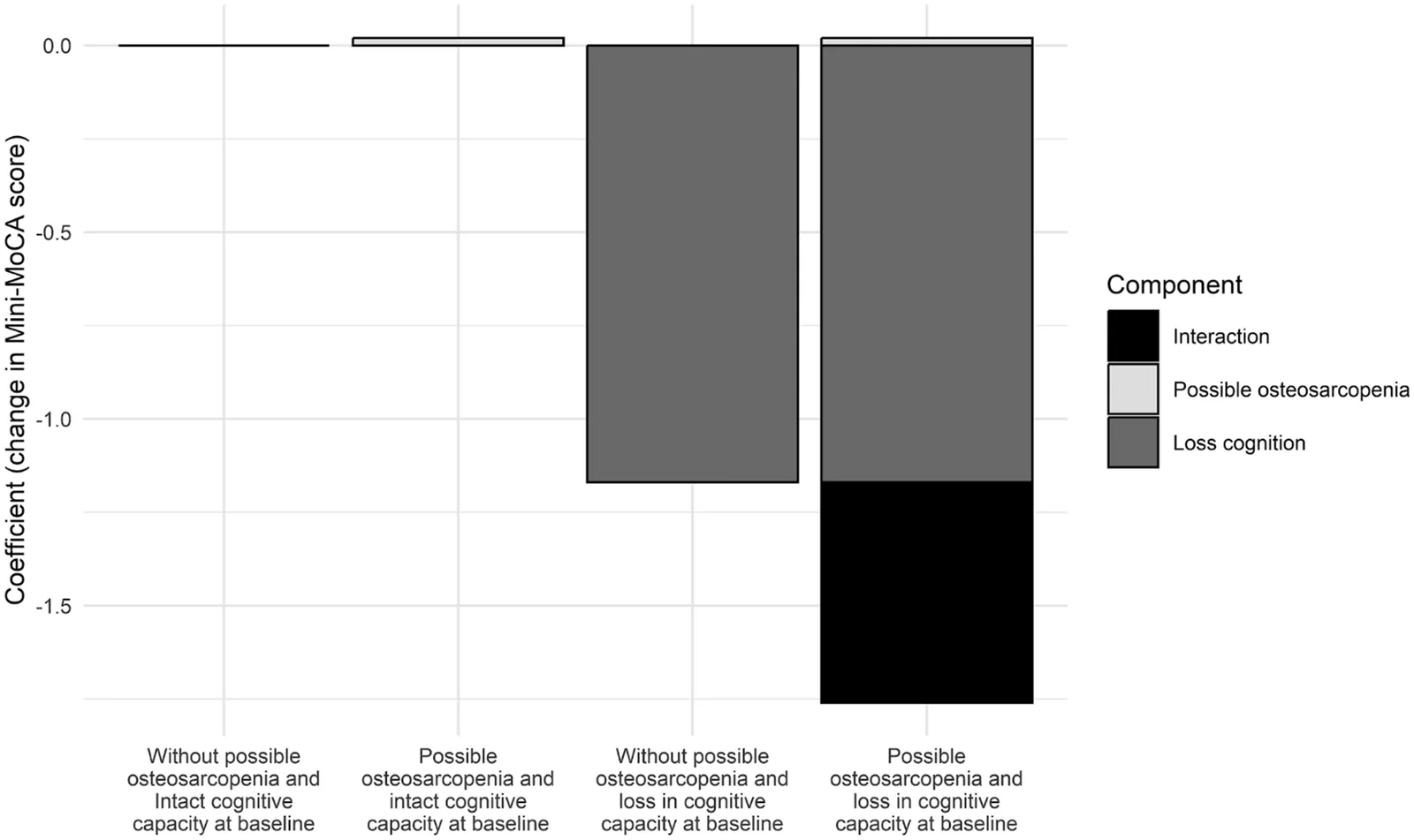
Summary of effect modification of baseline osteosarcopenia and cognition status on cognitive function 6 months post-baseline (*n* = 2047).

**Table 4:** Association between possible osteosarcopenia and cognitive function (measured by the mini-MoCA) as well as each domain at the 6-month follow-up (*n* = 2047).^a^

	Coefficient	95% CI	*p*-Value
**Total cognition function**			
**Osteosarcopenia (Ref: No possible osteosarcopenia)**			
Possible osteosarcopenia	0.02	(−0.37, 0.40)	.926
**Baseline cognition capacity (Ref: Intact cognitive capacity^b^)**			
Loss in cognitive capacity^b^	−1.17	(−1.52, −0.82)	<.001
**Osteosarcopenia#Baseline cognition capacity interaction (Ref: No possible osteosarcopenia#Intact cognitive capacity^c^)**			
Possible osteosarcopenia#Loss in cognitive capacity^b^	−0.59	(−1.16, −0.03)	.039
Age	−0.08	(−0.10, −0.06)	<.001
**Financial resources (Ref: More than adequate)**			
Adequate	−0.74	(−1.24, −0.23)	.005
Occasionally adequate	−1.27	(−1.86, −0.68)	<.001
Usually inadequate	−1.04	(−1.90, −0.17)	.020
**Number of chronic conditions (Ref: No)**			
1-3 chronic conditions	0.21	(−0.16, 0.58)	.265
≥4 chronic conditions	0.48	(−0.04, 0.92)	.032
**Frailty (Ref: Non-frail)**			
Pre- or intermediate frail	−0.12	(−0.53, 0.30)	.566
Frailty	−0.48	(0.04, 0.92)	.032
Instrumental activities of daily living	0.10	(0.02, 0.18)	.013
Lubben Social Network Scale	0.04	(0.02, 0.07)	<.001
**Delayed recall domain (*N = *1540)**			
**Osteosarcopenia (Ref: No osteosarcopenia)**			
Possible osteosarcopenia^c^	−0.01	(−0.06, 0.04)	.800
Osteosarcopenia*Baseline cognitive domain interaction (Ref: No osteosarcopenia# Cognitive intact^c^)			
Possible osteosarcopenia*Loss in cognitive capacity^b^	−0.41	(−0.47, −0.35)	<.001
**Orientation domain (*N = *1651)**			
**Osteosarcopenia (Ref: No osteosarcopenia)**			
Possible osteosarcopenia^d^	−0.03	(−0.05, 0.02)	.031
Osteosarcopenia*Baseline cognitive domain interaction (Ref: No osteosarcopenia# Cognitive intact^c^)			
Possible osteosarcopenia*Loss in cognitive capacity^b^	−0.12	(−0.15, −0.09)	<.001
**Language fluency domain (*N = *1542)**			
**Osteosarcopenia (Ref: No osteosarcopenia)**			
Possible osteosarcopenia^e^	−0.14	(−0.39, 0.10)	.242
Osteosarcopenia*Baseline cognitive domain interaction (Ref: No osteosarcopenia# Cognitive intact^c^)			
Possible osteosarcopenia*Loss in cognitive capacity^b^	0.09	(−0.15, 0.33)	.475

a Sample size includes 819 imputed cases.

b Loss in cognitive capacity: Montreal Cognitive Assessment (MoCA) score ≤25; Intact cognitive capacity: MoCA score ≥26.

c Adjusted for age, gender, financial resources, household type, living arrangement, frailty, Lubben social network, baseline cognitive capacity.

d Adjusted for age, financial resources, living arrangement, number of chronic conditions, instrumental activities of daily living, baseline cognitive capacity.

e Adjusted for age, ethnicity, highest educational level, financial resources, alcohol consumption, frailty, baseline cognitive capacity.

Additionally, when examining the delayed recall domain of the mini-MoCA, the interaction term between possible osteosarcopenia and cognitive capacity at baseline was statistically significant, indicating that the effect of possible osteosarcopenia on delayed recall scores was conditional on baseline cognitive capacity. Among those with loss in cognitive capacity at baseline, possible osteosarcopenia was associated with an additional 0.41-unit (over a score of 5 points) reduction in delayed recall scores. When comparing with those without possible osteosarcopenia and intact cognitive capacity at baseline, those with possible osteosarcopenia and loss in cognitive capacity was associated with an additional 0.42-unit (out of 5) loss in delayed recall scores (Table 4 and Figure S1). Similarly, the effect of possible osteosarcopenia on orientation domains was conditional on baseline cognitive capacity as well. The corresponding reductions in orientation scores were 0.12 units (out of 5) among those with baseline cognitive loss, and 0.15 units (out of 5) relative to those without possible osteosarcopenia and intact cognitive capacity at baseline (Table 4 and Figure S2). Interaction terms were not significant for language fluency domains.

Sensitivity analyses results are shown in Tables S16-S19. These tables show the complete case analysis for mini-MoCA and 3 domains after omitting participants who had imputed values. The results of these alternative specifications are largely consistent with the direction and magnitude of results from the main analyses. The interaction term between possible osteosarcopenia and baseline cognitive capacity for mini-MoCA and delayed recall domain remained significant, but it becomes nonsignificant for orientation and language fluency components in the sensitivity analysis.

## Discussion

This study aimed to assess the association between baseline possible osteosarcopenia and IC, with an emphasis on cognitive function at 6 months post-baseline. Through a series of multi-level mixed-effect models, possible osteosarcopenia was found to be associated with poorer IC overall and poorer cognitive capacity at baseline compared to those without possible osteosarcopenia. Among older adults with loss in cognitive capacity at baseline, possible osteosarcopenia at baseline predicted cognitive deficits at 6 months post-baseline, particularly in the delayed recall domain. However, there was no significant difference in cognitive function at 6 months post-baseline between older adults with and without possible osteosarcopenia if they had intact cognitive capacity at baseline.

This study found that the prevalence of possible osteosarcopenia in a national cohort of 2291 Singaporean older adults is 50.3%. This prevalence estimate is higher than the few studies examining osteosarcopenia prevalence in Singapore (11.7% to 12.9%).^46^^,^^47^ The higher estimates in our study likely reflect the variation in how osteosarcopenia has been operationalized. The definition used in this study, reflecting possible osteosarcopenia, differs from standard diagnostic criteria and may have increased likelihood of misclassification, including false positives. Osteoporosis and sarcopenia are best diagnosed using gold-standard imaging technologies, such as BMD *T*-scores and computed tomography (CT) or magnetic resonance imaging (MRI) for assessing muscle mass. However, only a proportion of participants in the TARGET cohort opted in for DXA scans, and CT/MRI modalities were not used. As such, due to data availability, possible osteoporosis and possible sarcopenia were defined using screening-based measures, including the OSTA index and AWGS2019-based handgrip strength thresholds, rather than diagnostic assessments of bone mineral density and muscle mass. While the possible osteosarcopenia classification showed acceptable sensitivity and specificity in the DXA subsample, these estimates should be interpreted cautiously due to potential selection bias, as participants in the subsample were voluntary and may be healthier.

### Osteosarcopenia and intrinsic capacity

Although there have not been sufficient high-quality publications on osteosarcopenia and IC, our results are consistent with existing studies exploring the relationships of osteoporosis and sarcopenia with IC and frailty.^48^^,^^49^ Some studies have demonstrated that older adults with poor handgrip strength experienced decline in IC scores, which may increase dependency on activities of daily living (ADLs) and IADLs, and reducing quality of life. This mechanism may be possible in Singapore, where older adults with possible osteosarcopenia may have poor IC, resulting in events such as falls, chronic conditions, and social isolation. Concurrently, education, better quality of life, and greater social networks were associated with greater IC scores in our study. A 13-year population-based study among older adults in the Netherlands showed how educational level was associated with frailty and demonstrated that factors such as multiple chronic conditions, insufficient income, and poor psychosocial outcomes attenuated the protective effect of education on frailty incidence.^50^

Our analysis suggests that osteosarcopenia is associated with a lower odds of intact capacity in locomotion, sensory, and psychological capacities, though not statistically significant. This may be due to the instruments used for data collection. While vitality and cognitive capacities were determined based on objective assessments (BMI and MoCA), sensory and psychological capacities were determined through self-reported questionnaires. Additionally, as gait speed was measured using a 5-min walk in the community setting, there may be larger variability in the gait data collected in real-world settings compared to clinical settings.^51^ Older adults may tend to walk slower due to their familiar settings or distractions in the environment.^52^ The cutoff score for gait speed, while well-established in the literature for fall prevention, is widely used in clinical settings. It also uses the same cutoff value for all participants regardless of sex and age. This single cutoff may have resulted in potential overestimation in the loss of locomotion capacity. Hence, using real-world gait data, while being more representative of gait in daily lives, may have contributed to the smaller than expected prevalence of intact locomotion capacity in our cohort.

### Osteosarcopenia and cognition

Beyond overall IC scores, we showed that older adults with possible osteosarcopenia had poorer cognition at baseline. Previous work has also explored the longitudinal association between sarcopenia and cognitive impairment with mixed results, potentially due to poor statistical power. A study of 131 Japanese community-dwelling older adults reported a significant association between sarcopenia and cognitive decline after a year.^53^

Our study shows that the relationship between possible osteosarcopenia at baseline and cognitive function at 6 months post-baseline was significant among those with impaired cognition at baseline, though this relationship was not observed among those with intact baseline cognitive capacity. This result likely reflects the 6-month follow-up period, which is a relatively short period of time to detect changes in cognitive function among cognitively healthy persons. As this cohort is still being followed up, future work is in the pipeline to analyze long-term trajectories in cognitive function over the two-year period. Nevertheless, we were still able to detect highly significant differences between those with and without osteosarcopenia for impaired cognition; demonstrating that osteosarcopenia may be an important factor in driving adverse outcomes in cognitive function.

Our findings highlighted that this longitudinal association was primarily driven by poor delayed recall ability. This has important clinical implications, as delayed recall ability reflects episodic memory capacity and is one of the first and most salient symptoms to deteriorate in preclinical symptoms of cognitive impairment.^54^ Delayed recall tests specifically engage the hippocampus and medial temporal lobe, regions which are vulnerable to systemic stressors such as inflammation, metabolic dysregulation, and reduced blood flow, often associated with osteosarcopenia.^55^ This effect may also be due to plausible shared pathways in inflammation and muscle-bone-brain axis.^56^ Additionally, findings from our work also demonstrated that the longitudinal association may also be driven by poor orientation ability. Orientation ability is one of the domains that often remain intact until further widespread neural changes occur.^57^ When examining the significant relationships of these domains together, this may suggest osteosarcopenia plays a role in both early cognitive impairment and gradual decline.

Multiple chronic conditions and polypharmacy among those with osteosarcopenia may also contribute to excessive oxidative stress and age-related chronic low-grade inflammation,^58^ resulting in downstream cognitive decline and musculoskeletal degeneration inherent in osteosarcopenia.^59^ However, the role of social determinants of health (SDOH) such as poor financial status and dependency in IADLs may be more influential in driving the development of osteosarcopenia and subsequent poor cognitive function. One study had shown that the effect of SDOH, indicating access to healthcare, was driving osteoporosis diagnosis, rather than the role of inflammation from chronic stress determined by high-sensitivity C-reactive proteins.^60^ Aligned with our findings, SDOHs that indicate access to resources and potential underlying inequalities were associated with osteosarcopenia and cognitive function. Further work is required to establish a causal mechanism between baseline osteosarcopenia status among those cognitively impaired, SDOHs, and subsequent cognitive decline.

In terms of other risk factors associated with cognition, older age was associated with poorer cognition scores at 6 months post-baseline, consistent with previous studies.^61^^,^^62^ Interestingly, greater chronic conditions were linked to better cognition at 6 months post-baseline, although most research reports the opposite.^63^ While some may argue that participants could plausibly have multiple but well-managed conditions, we acknowledge that disease severity, capability for self-management, and treatment quality were not measured in this study. This artifact observed may also be due to model overadjustment after multiple imputation.

More importantly, our results found ethnic differences in cognitive capacity. While some attribute this to variations in education,^64^ we believe that ethnic variations in cognitive performance across various studies in Singapore may speak to the need for a validated, ethnically tailored normative scoring system.^65^

### Implications

Given the evidence linking possible osteosarcopenia with poor IC domains, particularly that of cognition, from our study, prevention of osteosarcopenia may be crucial to prevent or slow the deterioration of both musculoskeletal and cognitive systems. Prevention strategies should be rooted on both early detection and multicomponent interventions. Systematic screening for osteosarcopenia later in life should be integrated into national chronic disease screening programs or recommendations. In Singapore, the Healthier SG initiative encourages annual health screenings from those above age 40, which may provide opportunities to include BMD assessments and physical performance tests. For those diagnosed with osteosarcopenia, further evaluations for cognitive impairment should be conducted, along with strategies to prevent cognitive decline. Similarly, those with preexisting cognitive impairment should be screened for low BMD and muscle loss. Additionally, given diagnostic limitations and costs, opportunistic rather than universal screening may be more feasible, particularly in individuals presenting with symptoms such as low mood, difficulties in daily activities, or recurrent falls.

Concurrently, more comprehensive strategies should take place to address overall musculoskeletal health and reducing opportunities of fracture. This may include progressive resistance training or multicomponent programs that include physical activity and optimized nutrition intake to increase muscle mass, muscle strength, and physical performance.^66^ Such interventions have been shown to prevent and/or delay cognitive impairment.^67^ Lastly, it is also important to reduce and prevent adverse SDOH, such as loneliness, limited social networks, and fear of falling, which are preventable and strongly associated with chronic stress and reduced cognitive engagement.^68^ Building resilience against stressors of social determinants of health should also be considered as potential prevention strategies of both osteosarcopenia and poorer cognition.

### Strengths and limitations

To our knowledge, this is the first large-scale, longitudinal study to examine osteosarcopenia in relation to overall IC and its cognition domain among community-dwelling older adults. However, several limitations to this work. As there is no standardized approach to calculating and defining IC, harmonizing these findings with others may be challenging. To mitigate this, the study applied definitions and methods established by others to align with current literature. In terms of the study design, we acknowledge that the study excluded individuals with severe cognitive impairment at baseline for safety reasons. As this skews the sample toward those who have better cognitive function at baseline, the effect of baseline possible osteosarcopenia on cognitive function at 6 months post-baseline may have been underestimated. Additionally, only half of the cohort had completed the mini-MoCA test at the 6-month follow-up, which may be a source of bias to in our study, as those with poorer health or greater impairment were more likely to decline participation or be unable to complete the test. Given that our results demonstrate a significant positive association between possible osteosarcopenia status and lower cognitive function at 6 months post-baseline, the inclusion of the aforementioned participants would have strengthened the observed relationship. The definition of possible osteosarcopenia relied on screening instruments rather than formal clinical diagnostic criteria, which may have introduced misclassification bias. Furthermore, existing validation studies of the OSTA have reported relatively low positive predictive values alongside high negative predictive values,^69^^,^^70^ indicative of a tendency toward overidentification of at-risk individuals (false positives). Moreover, we acknowledged that reverse causality cannot be ruled out, such that poor intrinsic capacity and/or cognitive impairment may drive the development of osteosarcopenia. Longer follow-up is required to track the temporal onset of these conditions to establish the direction of causality.

Findings from this study should be interpreted with these possibilities in mind. Finally, this longitudinal analysis is currently based on 6 months of follow-up, which may be too short to capture meaningful cognitive change in a relatively healthy sample. Ongoing work will build on these results to clarify the mechanism and implications of osteosarcopenia and subsequent cognitive function after 12 and even 24 months. Future work will also include linking this cohort with electronic medical records and monitoring diagnosis of cognitive impairment over time.

## Conclusion

In conclusion, possible osteosarcopenia was prevalent in more than 50% of a national cohort of community-dwelling older adults and was associated with loss in intrinsic capacity, most notably loss in the cognitive domain. Possible osteosarcopenia at baseline was predictive of poor cognitive function at 6 months post-baseline, particularly among those with preexisting loss in cognitive capacity. This was driven by the delayed recall domain, an early indicator of decline in episodic memory. These findings support routine osteosarcopenia screening and integration with cognitive assessments for early identification, routine screening, and management of bone and muscle health among populations with cognitive impairment.

## Supplementary Material

glag211_Supplementary_Data

## Data Availability

Data utilized for this study are available from the Centre for Ageing Research & Education (CARE). De-identified data can be made available upon reasonable request and permission from CARE.
