# Supplementary material for: Possible osteosarcopenia is associated with loss in intrinsic capacity and early cognitive deficits: a longitudinal study of older adults in Singapore
Source: J Gerontol A Biol Sci Med Sci. 2026 Aug 26;81(11):glag211. doi: 10.1093/gerona/glag211 (PMC13641826; doi:10.1093/gerona/glag211)
Supplement: Supplementary file 1 [file glag211_supplementary_data.docx]

## **Supplementary Text**

**Methods**

## **Study Design**

Data were analysed from the Targeted Assessment and Recruitment of Geriatrics for Effective fall prevention Treatments (TARGET) study, an ongoing national prospective cohort study of n=2,291 community-dwelling Singapore residents aged ≥60 years. Participants were recruited between October 2022 and October 2024. After study enrolment, participants underwent a comprehensive baseline fall risk assessment (T0) followed by eight follow-up assessments (T1-T8) every three months for two years to prospectively monitor their fall, fracture and cognitive status. Ethics approval for the study was granted by the National University of Singapore Institutional Review Board (NUS-IRB-2021-168). All aspects of study reporting conformed to the STROBE guidelines (Supplementary Table 1).

## **Participants and Setting**

Participants were drawn from a nationally representative multistage random sample of 7,501 households with at least one older adult resident obtained from the Department of Statistics of Singapore. Addresses with predominant Malay or Indian residents, or with at least one resident aged ≥ 75 years were oversampled by a factor of two. Trained field interviewers conducted home visits to recruit eligible participants from the sampling frame. Inclusion criteria included: 1) Singapore citizen or permanent resident, 2) aged 60-105 years old, 3) spoke at least one of the four official languages (English, Mandarin, Malay, or Tamil), 4) ambulatory, 5) able to see with or without glasses, and 6) able to hear with or without hearing aids. Individuals with one or more of the following were excluded for safety reasons: 1) Abbreviated Mental Test score <6, 2) blood pressure outside of normal range (defined as ≥200/110 mmHg, or ≥180/100 mmHg AND heart rate >120/minute, or ≤90/60 mmHg), 3) symptoms of chest discomfort, breathlessness, dizziness, or profuse sweating at the time of recruitment, or 4) acute cardiac event in the past 30 days.

## **Data Collection**

All consenting participants underwent a 2-hour home-based baseline questionnaire including an anthropometric and gait assessment conducted by trained field interviewers. This interview comprises of (i) a structured interview to ascertain sociodemographic, environmental, clinical, functional, cognitive, and psychosocial status, and (ii) a five-minute walk test to assess gait while wearing ZurichMOVE inertial measurement unit (IMU) motion sensors, at home or a place of convenience^1^. The IMU sensors were placed on six designated anatomical locations (feet, wrists, trunk and head) to acquire gait data using accelerometer and gyroscopes, enabling us to collect gait data^2^. Participants who reported no history of bilateral hip replacement were then invited for a clinic visit at the Singapore Eye Research Institute for a Dual Energy X-ray Absorptiometry (DXA) scan to assess bone mineral density. Participation for this scan was completely voluntary, via an opt-in basis, resulting in only a proportion of participants undergoing the DXA scans (895/2,291, 39.1%). Following baseline assessment, participants were prospectively followed up via telephone every three months for a period of two years to determine if they have experienced a fall and to assess their cognitive status.

## **Sample Size and Power Analysis**

To ensure our study was sufficiently powered, a sample size calculation was conducted using G*Power^45^, modelling linear multiple regressions. Assuming a small effect size of f^2^ = 0.02, α = 0.05, power (1-β) = 0.80 and the greatest number of predictors = 8 predictors, we obtained an a priori sample size of 759 participants. To account for the clustering effect of participants within a household, the sample size was multiplied with a design effect (DE) of 1.128. The DE was obtained based on the average household cluster size of 1.4, and an ICC of 0.322 at the household level. The resulting sample size required for a well-powered analysis is 857 participants, demonstrating that our analysis of 1,228 participants was sufficiently powered.

**References**

1.	Kim YK, Pai SGS, Choi JO, et al. Leveraging Deep Learning and Wearables for Automatically Identifying Gait Event: Effects of Age and Location of Sensors on the Assessment of Gait Events. *IEEE Sensors Journal*. 2025;25(1):792-802. doi:10.1109/JSEN.2024.3487018

2.	Iosa M, Picerno P, Paolucci S, Morone G. Wearable inertial sensors for human movement analysis. *Expert Review of Medical Devices*. 2016/07/02 2016;13(7):641-659. doi:10.1080/17434440.2016.1198694

**Supplementary Table 1: STROBE Statement—checklist of items that should be included in reports of observational studies**

|  | **Item No** | **Recommendation** | **Page no** |
| --- | --- | --- | --- |
| **Title and abstract** | 1 | (*a*) Indicate the study’s design with a commonly used term in the title or the abstract | 1, 3 |
|  |  | (*b*) Provide in the abstract an informative and balanced summary of what was done and what was found | 3 |
| **Introduction** | | |  |
| Background/rationale | 2 | Explain the scientific background and rationale for the investigation being reported | 5 - 6 |
| Objectives | 3 | State specific objectives, including any prespecified hypotheses | 7 |
| **Methods** | | |  |
| Study design | 4 | Present key elements of study design early in the paper | 7 |
| Setting | 5 | Describe the setting, locations, and relevant dates, including periods of recruitment, exposure, follow-up, and data collection | 8 |
| Participants | 6 | (*a*) *Cohort study*—Give the eligibility criteria, and the sources and methods of selection of participants. Describe methods of follow-up  *Case-control study*—Give the eligibility criteria, and the sources and methods of case ascertainment and control selection. Give the rationale for the choice of cases and controls  *Cross-sectional study*—Give the eligibility criteria, and the sources and methods of selection of participants | Supplementary Text |
|  |  | (*b*) *Cohort study*—For matched studies, give matching criteria and number of exposed and unexposed  *Case-control study*—For matched studies, give matching criteria and the number of controls per case |  |
| Variables | 7 | Clearly define all outcomes, exposures, predictors, potential confounders, and effect modifiers. Give diagnostic criteria, if applicable | 8 - 12 |
| Data sources/ measurement | 8* | For each variable of interest, give sources of data and details of methods of assessment (measurement). Describe comparability of assessment methods if there is more than one group | 8 – 12, Supplementary Table 5 |
| Bias | 9 | Describe any efforts to address potential sources of bias | Not applicable |
| Study size | 10 | Explain how the study size was arrived at | 14 |
| Quantitative variables | 11 | Explain how quantitative variables were handled in the analyses. If applicable, describe which groupings were chosen and why | 8 - 12 |
| Statistical methods | 12 | (*a*) Describe all statistical methods, including those used to control for confounding | 12 - 14 |
|  |  | (*b*) Describe any methods used to examine subgroups and interactions | 13 |
|  |  | (*c*) Explain how missing data were addressed | Not applicable |
|  |  | (*d*) *Cohort study*—If applicable, explain how loss to follow-up was addressed  *Case-control study*—If applicable, explain how matching of cases and controls was addressed  *Cross-sectional study*—If applicable, describe analytical methods taking account of sampling strategy | Not applicable |
|  |  | (*e*) Describe any sensitivity analyses | 9 |

*Give information separately for cases and controls in case-control studies and, if applicable, for exposed and unexposed groups in cohort and cross-sectional studies.

Note: An Explanation and Elaboration article discusses each checklist item and gives methodological background and published examples of transparent reporting. The STROBE checklist is best used in conjunction with this article (freely available on the Web sites of PLoS Medicine at http://www.plosmedicine.org/, Annals of Internal Medicine at http://www.annals.org/, and Epidemiology at http://www.epidem.com/). Information on the STROBE Initiative is available at [www.strobe-statement.org](https://www.strobe-statement.org).

**Supplementary Table 2: Diagnostic criteria for osteosarcopenia**

| **Condition** | **Measurement Method** | **Diagnostic Criteria** |
| --- | --- | --- |
| **Osteoporosis** | DXA BMD T-scores | T-score ≤ –2.5 for either the total hip, femoral neck or lumbar spine |
| **Sarcopenia** | Muscle mass (whole-body DXA) | Low muscle mass: appendicular lean mass (ALM) divided by height squared; (ALM/height²) < 7.0 kg/m² (men) or < 5.4 kg/m² (women) |
|  | Muscle strength (handgrip dynamometer) | Low muscle strength: maximum grip strength < 28 kg (men) or < 18 kg (women) |
| **Osteosarcopenia** | Presence of both osteoporosis and sarcopenia as per diagnostic criteria above | |

DXA: Dual-energy X-ray absorptiometry
BMD: Bone mineral density

**Supplement Table 3: Diagnostic performance of possible osteosarcopenia classifications comparing with gold-standard definitions based on bone mineral density, muscle mass and muscle strength** ^¶^

| **Measure** | **Estimate** | **95% CI** |
| --- | --- | --- |
| Prevalence | 22.7% | 19.2 – 26.6% |
| Sensitivity | 92.3% | 85.9 – 96.4% |
| Specificity | 71.4% | 66.6 – 75.8% |
| ROC area | 0.82 | 0.79 – 0.85 |
| Positive predictive value | 48.6% | 41.9 – 55.4% |
| Negative predictive value | 96.9% | 94.2 – 98.6% |

CI: confidence interval

ROC: Receiver Operating Characteristic

¶: Dianostic performance of osteosarcopenia were calculated based on 515 participants. Data on muscle mass was available for 2,283 participants (8 people did not proceed with handgrip strength assessment due to safety concerns). Of this group, 515 participants opted for the DXA scans.

**Supplementary Table 4: Differences in baseline characteristics of study participants who completed mini-MoCA and those who did not complete mini-MoCA at the 6-month follow-up (n = 2,291)**

| **Characteristic** | **Total**  **(N = 2,291)** | **Participants completed mini-MOCA test**  **(N = 1,228)** | **Participants did not complete mini-MOCA test**  **(N = 1,063)** | **p-value** |
| --- | --- | --- | --- | --- |
| **Age (Mean, SD)** | 74.6 (7.9) | 73.6 (7.5) | 75.6 (8.1) | <0.001 |
| **Age (N, %)** |  |  |  | <0.001 |
| 60-64 | 302 (13.2) | 174 (14.2) | 128 (12.0) |  |
| 65-69 | 388 (16.9) | 245 (20.0) | 143 (13.5) |  |
| 70-74 | 362 (15.8) | 207 (16.9) | 155 (14.6) |  |
| 75-79 | 614 (26.8) | 329 (26.8) | 285 (26.8) |  |
| 80-84 | 380 (16.6) | 180 (14.7) | 200 (18.8) |  |
| 85+ | 245 (10.7) | 93 (7.6) | 152 (14.3) |  |
| **Gender (N, %)** |  |  |  | 0.054 |
| Male | 964 (42.1) | 494 (40.2) | 470 (44.2) |  |
| Female | 1,327 (57.9) | 734 (59.8) | 593 (55.8) |  |
| **Ethnicity (N, %)** |  |  |  | <0.001 |
| Chinese | 1,521 (66.4) | 872 (71.0) | 649 (61.1) |  |
| Malay | 433 (18.9) | 197 (16.0) | 236 (22.2) |  |
| Indian | 290 (12.7) | 143 (11.6) | 147 (13.8) |  |
| Other | 47 (2.1) | 16 (1.3) | 31 (2.9) |  |
| **Highest educational level (N, %)** |  |  |  | <0.001 |
| No formal education | 367 (16.0) | 154 (12.5) | 213 (20.0) |  |
| Primary | 741 (32.3) | 357 (29.1) | 384 (36.1) |  |
| Secondary | 757 (33.0) | 435 (35.4) | 322 (30.3) |  |
| Vocational or Institute of Technical Education (ITE) | 44 (1.9) | 27 (2.2) | 17 (1.6) |  |
| Junior College (JC) or GCE ‘A’ Level | 138 (6.0) | 86 (7.0) | 52 (4.9) |  |
| Polytechnic | 93 (4.1) | 57 (4.6) | 36 (3.4) |  |
| University and above | 151 (6.6) | 112 (9.1) | 39 (3.7) |  |
| **Financial resources (N, %) **** |  |  |  | <0.001 |
| More than adequate | 72 (3.1) | 56 (4.6) | 16 (1.5) |  |
| Adequate | 1,731 (75.6) | 932 (75.9) | 799 (75.2) |  |
| Occasionally adequate | 400 (17.5) | 198 (16.1) | 202 (19.0) |  |
| Usually inadequate | 83 (3.6) | 41 (3.3) | 42 (3.6) |  |
| **Housing Categories (N, %)** |  |  |  | 0.017 |
| HDB flat (1-2 room) | 258 (11.3) | 125 (10.2) | 133 (12.5) |  |
| HDB flat (3-4 rooms) | 1,387 (60.5) | 729 (59.4) | 658 (61.9) |  |
| HDB flat (5 room and above) and Private housing/others | 646 (28.2) | 374 (30.5) | 272 (25.6) |  |
| **Living Arrangements (N, %)** |  |  |  | <0.001 |
| Living Alone | 243 (10.6) | 159 (13.0) | 84 (7.9) |  |
| Living with Child and Spouse | 699 (30.5) | 359 (29.2) | 340 (32.0) |  |
| Living with Child Only | 350 (15.3) | 162 (13.2) | 188 (17.7) |  |
| Living with Spouse Only | 719 (31.4) | 383 (31.2) | 336 (31.6) |  |
| Living with Others Only (Include Foreign Domestic Worker) | 280 (12.2) | 165 (13.4) | 115 (10.8) |  |
| **Intrinsic capacity score (Mean, SD)** | 2.8 (1.0) | 2.4 (0.9) | 2.07 (0.9) | <0.001 |
| **Locomotion score (N, %) **** |  |  |  | <0.001 |
| **0** | 1,903 (83.1) | 1,003 (81.7) | 900 (84.7) |  |
| **1** | 248 (10.8) | 166 (13.5) | 82 (7.7) |  |
| **Vitality score (N, %) **** |  |  |  | 0.024 |
| **0** | 139 (6.1) | 65 (5.3) | 74 (7.0) |  |
| **1** | 2,148 (93.9) | 1,163 (94.7) | 985 (92.7) |  |
| **Cognition score (N, %) **** |  |  |  | <0.001 |
| **0** | 1,364 (59.5) | 623 (50.7) | 741 (69.7) |  |
| **1** | 926 (40.4) | 604 (49.2) | 322 (30.3) |  |
| **Psychological score (N, %) **** |  |  |  | 0.077 |
| **0** | 176 (7.7) | 80 (6.5) | 96 (9.0) |  |
| **1** | 2,111 (92.1) | 1,146 (93.3) | 965 (90.8) |  |
| **Sensory score (N, %)** |  |  |  | <0.001 |
| **0** | 597 (26.1) | 283 (23.1) | 314 (29.5) |  |
| **1** | 1694 (73.9) | 945 (77.0) | 749 (70.5) |  |
| **Current smoker (N, %) **** |  |  |  | 0.469 |
| No | 2,117 (92.4) | 1,139 (92.8) | 978 (92.0) |  |
| Yes | 173 (7.6) | 89 (7.3) | 84 (7.9) |  |
| **Alcohol consumption status (N, %) **** |  |  |  | <0.001 |
| Currently consume | 293 (12.8) | 202 (16.5) | 91 (8.6) |  |
| Consumed in the past | 193 (8.4) | 96 (7.8) | 97 (9.1) |  |
| Never consumed | 1,804 (78.7) | 929 (75.7) | 875 (82.3) |  |
| **Polypharmacy (N, %)** |  |  |  | <0.001 |
| No | 1,717 (75.0) | 959 (78.1) | 758 (71.3) |  |
| Yes | 574 (25.1) | 269 (21.9) | 305 (28.7) |  |
| **Taking glucocorticoids (N, %) **** |  |  |  | 0.711 |
| No | 2,270 (99.1) | 1,215 (98.9) | 1,055 (99.3) |  |
| Yes | 14 (0.6) | 9 (0.7) | 5 (0.5) |  |
| **Taking calcium or Vitamin D supplementation (N, %) **** |  |  |  | 0.340 |
| No | 1,274 (55.6) | 666 (54.2) | 608 (57.2) |  |
| Yes | 1,005 (43.9) | 556 (45.3) | 449 (42.2) |  |
| **Taking medication to improve mood (N, %) **** |  |  |  | 0.241 |
| No | 2,257 (98.5) | 1,214 (98.9) | 1,043 (98.1) |  |
| Yes | 33 (1.4) | 14 (1.1) | 19 (1.8) |  |
| **Taking medication for heart rate (N, %)** |  |  |  | 0.279 |
| No | 2,062 (90.0) | 1,113 (90.6) | 949 (89.3) |  |
| Yes | 229 (10.0) | 115 (9.4) | 114 (10.7) |  |
| **Self-reported diabetes status (N, %)** |  |  |  | 0.006 |
| No | 1,593 (69.5) | 884 (72.0) | 709 (66.7) |  |
| Yes | 698 (30.5) | 344 (28.0) | 354 (33.3) |  |
| **Self-reported hyperlipidaemia status (N, %)** |  |  |  | 0.669 |
| No | 903 (39.4) | 489 (39.8) | 414 (39.0) |  |
| Yes | 1388 (60.6) | 739 (60.2) | 649 (61.1) |  |
| **Self-reported hypertension status (N, %)** |  |  |  | 0.036 |
| No | 904 (39.5) | 509 (41.5) | 395 (37.2) |  |
| Yes | 1387 (60.6) | 719 (58.6) | 668 (62.8) |  |
| **Physical activity levels (N, %) **** |  |  |  | 0.002 |
| Low level | 749 (32.7) | 360 (29.3) | 389 (36.6) |  |
| Moderate level | 1,134 (49.5) | 632 (51.5) | 502 (47.2) |  |
| High level | 389 (17.0) | 224 (18.2) | 165 (15.5) |  |
| **Resilience Scale (Mean, SD)** | 3.8 (0.5) | 3.8 (0.5) | 3.8 (0.5) | 0.335 |
| **Number of chronic conditions (N, %)** |  |  |  | 0.713 |
| 0 chronic conditions | 302 (13.2) | 166 (13.5) | 136 (12.8) |  |
| 1-3 chronic conditions | 1,398 (61.0) | 753 (61.3) | 645 (60.7) |  |
| ≥ 4 chronic conditions | 591 (25.8) | 309 (25.2) | 282 (26.5) |  |
| **IADL (Mean, SD)** | 14.7 (2.6) | 15.2 (2.00) | 14.2 (3.2) | <0.001 |
| **EQ_5D Quality of Life (Mean, SD)** | 0.9 (0.6) | 0.9 (0.1) | 0.9 (0.2) | <0.001 |
| **Number of medications (Mean, SD)** | 3.8 (2.6) | 3.6 (2.5) | 4.0 (2.6) | <0.001 |
| **ICON-Falls Efficacy Scale (Fear of Falling) (Mean, SD)** | 17.0 (7.0) | 16.2 (6.3) | 18.0 (7.5) | <0.001 |
| **Lubben social network scale (Mean, SD)** | 13.8 (5.7) | 14.2 (5.8) | 13.2 (5.5) | <0.001 |
| **Loneliness score (Mean, SD)** | 3.6 (1.3) | 3.7 (1.3) | 3.6 (1.2) | 0.009 |
| **Number of insomnia symptoms (Mean, SD)** | 1.7 (1.6) | 1.7 (1.5) | 1.6 (1.6) | 0.637 |
| **Frailty (N, %) **** |  |  |  | <0.001 |
| Non-frail | 144 (6.3) | 99 (8.1) | 45 (4.2) |  |
| Pre- or intermediate frail | 1,525 (66.6) | 851 (69.3) | 674 (63.4) |  |
| Frailty | 390 (17.0) | 177 (14.4) | 213 (20.0) |  |
| **Appendicular Lean Mass (Mean, SD) **** | 16.1 (3.9) | 16.4 (4.1) | 16.0 (3.8) | 0.164 |
| **Sarcopenia (N, %) **** |  |  |  | <0.001 |
| **No sarcopenia** | 854 (37.0) | 326 (30.1) | 528 (43.0) |  |
| **Possible sarcopenia** | 1,429 (61.9) | 733 (69.0) | 696 (56.7) |  |
| **Osteoporosis (N, %) **** |  |  |  | <0.001 |
| **No possible osteoporosis** | 648 (28.1) | 283 (26.6) | 365 (29.7) |  |
| **Possible osteoporosis** | 1,640 (71.1) | 777 (73.1) | 863 (70.3) |  |

** Contains a missing value, thus percentages might not add up to 100%.

IADL: Independent activities of daily living

**Supplementary Table 5: Details of covariates used in analysis, with relevant literature justifying the use of covariates in the analysis.**

| **Type** | **Covariates** | **Literature** |
| --- | --- | --- |
| **Continuous** | Age | Huang, T., Li, C., Chen, F., Xie, D., Yang, C., Chen, Y., ... & Zheng, F. (2023). Prevalence and risk factors of osteosarcopenia: a systematic review and meta-analysis. BMC geriatrics, 23(1), 369.  <https://doi.org/10.1186/s12877-023-04085-9>  Wei, X., Chen, Y., Qin, J., Yang, Y., Yang, T., Yan, F., Zhang, Z., Han, L., & Ma, Y. (2024). Factors associated with the intrinsic capacity in older adults: A scoping review. Journal of clinical nursing, 33(5), 1739–1750. https://doi.org/10.1111/jocn.17017 |
|  | Number of chronic conditions | Inoue, T., Maeda, K., Nagano, A., Shimizu, A., Ueshima, J., Murotani, K., ... & Tsubaki, A. (2021). Related factors and clinical outcomes of osteosarcopenia: a narrative review. Nutrients, 13(2), 291.  <https://doi.org/10.3390/nu13020291>  Wei, X., Chen, Y., Qin, J., Yang, Y., Yang, T., Yan, F., Zhang, Z., Han, L., & Ma, Y. (2024). Factors associated with the intrinsic capacity in older adults: A scoping review. Journal of clinical nursing, 33(5), 1739–1750. https://doi.org/10.1111/jocn.17017 |
|  | Number of Medications | Tan, Y.Q., Ng, D.X., Gunasekaran, K. et al. Clinical characteristics and risk factors of osteoporosis among older Asian men with type-2 diabetes mellitus, hypertension, or hyperlipidaemia. (2024). Arch Osteoporos 19, 83. <https://doi.org/10.1007/s11657-024-01442-y>  Yu, X., Qian, Y., Zhang, Y., Chen, Y., & Wang, M. (2024). Association between polypharmacy and cognitive impairment in older adults: A systematic review and meta-analysis. Geriatric nursing (New York, N.Y.), 59, 330–337. <https://doi.org/10.1016/j.gerinurse.2024.07.005>  Meng, L. C., Hsiao, F. Y., Huang, S. T., Lu, W. H., Peng, L. N., & Chen, L. K. (2022). Intrinsic Capacity Impairment Patterns and their Associations with Unfavorable Medication Utilization: A Nationwide Population-Based Study of 37,993 Community-Dwelling Older Adults. The journal of nutrition, health & aging, 26(10), 918–925. https://doi.org/10.1007/s12603-022-1847-z |
|  | Instrumental Activities of Daily Living (IADL) | López-Teros, M. T., Rosas-Carrasco, O., Sánchez-García, S., Castro-Porras, L., Luna-López, A., & Agudelo-Botero, M. (2021). The association of osteosarcopenia with functional disability in community-dwelling Mexican adults 50 and older. Frontiers in Medicine, 8, 674724.  <https://doi.org/10.1016/j.jnha.2024.100368>  Makino, K., Lee, S., Bae, S., Shinkai, Y., Chiba, I., & Shimada, H. (2020). Relationship between instrumental activities of daily living performance and incidence of mild cognitive impairment among older adults: A 48-month follow-up study. Archives of gerontology and geriatrics, 88, 104034. <https://doi.org/10.1016/j.archger.2020.104034>  Ahmadi, S., Afshar, P. F., Malakouti, K., & Azadbakht, M. (2025). The relationship between intrinsic capacity and functional ability in older adults. BMC geriatrics, 25(1), 57. https://doi.org/10.1186/s12877-025-05709-y |
|  | EQ5D Quality of Life | Keramidaki, K., Tsagari, A., Hiona, M., & Risvas, G. (2019). Osteosarcopenic obesity, the coexistence of osteoporosis, sarcopenia and obesity and consequences in the quality of life in older adults≥ 65 years-old in Greece. Journal of frailty, sarcopenia and falls, 4(4), 91.  <https://www.jfsf.eu/article/4/91>  Stites, S. D., Harkins, K., Rubright, J. D., & Karlawish, J. (2018). Relationships Between Cognitive Complaints and Quality of Life in Older Adults With Mild Cognitive Impairment, Mild Alzheimer Disease Dementia, and Normal Cognition. Alzheimer disease and associated disorders, 32(4), 276–283. https://doi.org/10.1097/WAD.0000000000000262 |
|  | ICON-Falls Efficacy Scale (Fear of Falling) | Aibar-Almazán, A., Martínez-Amat, A., Cruz-Díaz, D., Jiménez-García, J. D., Achalandabaso, A., Sánchez-Montesinos, I., ... & Hita-Contreras, F. (2018). Sarcopenia and sarcopenic obesity in Spanish community-dwelling middle-aged and older women: Association with balance confidence, fear of falling and fall risk. Maturitas, 107, 26-32.  <https://doi.org/10.1016/j.maturitas.2017.10.001>  Vo, T.H.M., Nakamura, K., Seino, K. et al. (2020). Fear of falling and cognitive impairment in elderly with different social support levels: findings from a community survey in Central Vietnam. BMC Geriatr 20, 141. https://doi.org/10.1186/s12877-020-01533-8 |
|  | Lubben Social Networks Scale | Inoue, T., Maeda, K., Satake, S., Matsui, Y., & Arai, H. (2022). Osteosarcopenia, the co-existence of osteoporosis and sarcopenia, is associated with social frailty in older adults. Aging clinical and experimental research, 34(3), 535-543.  <https://doi.org/10.1007/s40520-021-01968-y>  Cheong, G., Tov, W., Choo, R. W. M., Tan, M., Lau, L. K., Lim, W. S., Ding, Y. Y., & Straughan, P. T. (2025). Exploring the relationship between intrinsic capacity and social participation in healthy ageing: Evidence from Singapore. The journal of nutrition, health & aging, 29(5), 100524. https://doi.org/10.1016/j.jnha.2025.100524 |
|  | Loneliness score | Inoue, T., Maeda, K., Satake, S., Matsui, Y., & Arai, H. (2022). Osteosarcopenia, the co-existence of osteoporosis and sarcopenia, is associated with social frailty in older adults. Aging clinical and experimental research, 34(3), 535-543.  <https://doi.org/10.1007/s40520-021-01968-y>  Guarnera, J., Yuen, E., & Macpherson, H. (2023). The Impact of Loneliness and Social Isolation on Cognitive Aging: A Narrative Review. Journal of Alzheimer's disease reports, 7(1), 699–714. https://doi.org/10.3233/ADR-230011 |
|  | Number of Insomnia Symptoms | Pourmotabbed, A., Ghaedi, E., Babaei, A., Mohammadi, H., Khazaie, H., Jalili, C., ... & Miraghajani, M. (2020). Sleep duration and sarcopenia risk: a systematic review and dose-response meta-analysis. Sleep and Breathing, 24(4), 1267-1278.  <https://doi.org/10.1007/s11325-019-01965-6>  Baril, A. A., Beiser, A. S., Sanchez, E., Mysliwiec, V., Redline, S., Gottlieb, D. J., O'Connor, G. T., Gonzales, M. M., Himali, D., Seshadri, S., Himali, J. J., & Pase, M. P. (2022). Insomnia symptom severity and cognitive performance: Moderating role of APOE genotype. Alzheimer's & dementia : the journal of the Alzheimer's Association, 18(3), 408–421. https://doi.org/10.1002/alz.12405 |
|  | Psychological Resilience score | Lee, S. Y., Tung, H. H., Peng, L. N., Chen, L. K., Hsu, C. I., & Huang, Y. L. (2020). Resilience among older cardiovascular disease patients with probable sarcopenia. Archives of gerontology and geriatrics, 86, 103939. https://doi.org/10.1016/j.archger.2019.103939  Du, C., Shen, Z., Mansoor, M., Chen, Q., & Katz, B. (2025). Psychological Resilience, Cognitive Function, and Physical Activity: A Longitudinal Mediation Study. The American journal of geriatric psychiatry : official journal of the American Association for Geriatric Psychiatry, 33(8), 901–910. https://doi.org/10.1016/j.jagp.2025.01.009 |
| **Categorical** | Gender (male/female) | Huang, T., Li, C., Chen, F., Xie, D., Yang, C., Chen, Y., ... & Zheng, F. (2023). Prevalence and risk factors of osteosarcopenia: a systematic review and meta-analysis. BMC geriatrics, 23(1), 369.  https://doi.org/10.1186/s12877-023-04085-9 |
|  | Ethnicity (Chinese/Malay/Indian/Other) | Heng, M.W.Y., Chan, A.W.D., Man, R.E.K. et al. (2023). Individual and combined associations of sarcopenia, osteoporosis and obesity with frailty in a multi-ethnic asian older adult population. BMC Geriatr 23, 802 <https://doi.org/10.1186/s12877-023-04500-1>  Sahadevan, S., Saw, S. M., Gao, W., Tan, L. C., Chin, J. J., Hong, C. Y., & Venketasubramanian, N. (2008). Ethnic differences in Singapore's dementia prevalence: the stroke, Parkinson's disease, epilepsy, and dementia in Singapore study. Journal of the American Geriatrics Society, 56(11), 2061–2068. <https://doi.org/10.1111/j.1532-5415.2008.01992.x>  Salinas-Rodríguez, A., Fernández-Niño, J.A., Rivera-Almaraz, A. et al. (2024). Intrinsic capacity trajectories and socioeconomic inequalities in health: the contributions of wealth, education, gender, and ethnicity. Int J Equity Health 23, 48. https://doi.org/10.1186/s12939-024-02136-0 |
|  | Education (no formal/primary/secondary/vocational or ITE/junior college or GCE ‘A’ level/polytechnic/university and above) | Silveira, E. A., Vinícius-Souza, G., Pereira, C. C., de Oliveira, C., Noll, M., & Pagotto, V. (2023). Osteosarcopenia later in life: prevalence and associated risk factors. Clinical Nutrition ESPEN, 58, 213-220.  <https://doi.org/10.1016/j.clnesp.2023.08.030>  Seblova, D., Berggren, R., & Lövdén, M. (2020). Education and age-related decline in cognitive performance: Systematic review and meta-analysis of longitudinal cohort studies. Ageing research reviews, 58, 101005. <https://doi.org/10.1016/j.arr.2019.101005>  Jiang, X., Chen, F., Yang, X., Yang, M., Zhang, X., Ma, X., & Yan, P. (2023). Effects of personal and health characteristics on the intrinsic capacity of older adults in the community: a cross-sectional study using the healthy aging framework. BMC geriatrics, 23(1), 643. https://doi.org/10.1186/s12877-023-04362-7 |
|  | Financial resources (more than adequate/adequate/occasionally adequate/usually inadequate)  Household type (HDB 1–2 rooms/HDB 3–4 rooms/HDB 5 rooms or above/private) | Wei, X., Chen, Y., Qin, J., Yang, Y., Yang, T., Yan, F., Zhang, Z., Han, L., & Ma, Y. (2024). Factors associated with the intrinsic capacity in older adults: A scoping review. Journal of clinical nursing, 33(5), 1739–1750. <https://doi.org/10.1111/jocn.17017>  Huang, Z., Lai, E. T. C., Hashimoto, H., Marmot, M., & Woo, J. (2025). Life-course socioeconomic inequalities, social mobility and healthy aging in older adults: A multi-cohort study. Archives of gerontology and geriatrics, 133, 105829. <https://doi.org/10.1016/j.archger.2025.105829> |
|  | Living Arrangements (alone/with children and spouse/with children only/with spouse only/with others) | Hu, P., Zhang, D., Wong, S. Y. S., Woo, J., Yu, R., Yip, B. H. K., & Poon, P. K. M. (2023). The Effect of Social Isolation on Sarcopenia: A Longitudinal Study among the Middle-Aged and Older Population in China. Gerontology, 69(6), 748–756. https://doi.org/10.1159/000529443  Yu, Y., Lv, J., Liu, J. et al. (2022). Association between living arrangements and cognitive decline in older adults: A nationally representative longitudinal study in China. BMC Geriatr 22, 843. <https://doi.org/10.1186/s12877-022-03473-x>  Jiang, X., Chen, F., Yang, X., Yang, M., Zhang, X., Ma, X., & Yan, P. (2023). Effects of personal and health characteristics on the intrinsic capacity of older adults in the community: a cross-sectional study using the healthy aging framework. BMC geriatrics, 23(1), 643. https://doi.org/10.1186/s12877-023-04362-7 |
|  | Current Smoking Status (yes/no) | Liu, Y., Hao, Q., Zhou, J., & Wu, J. (2024). A comprehensive meta-analysis of risk factors associated with osteosarcopenic obesity: a closer look at gender, lifestyle and comorbidities. Osteoporosis International, 35(5), 759-773.  <https://doi-org.libproxy1.nus.edu.sg/10.1007/s00198-023-07007-y>  Wei, X., Chen, Y., Qin, J., Yang, Y., Yang, T., Yan, F., Zhang, Z., Han, L., & Ma, Y. (2024). Factors associated with the intrinsic capacity in older adults: A scoping review. Journal of clinical nursing, 33(5), 1739–1750. https://doi.org/10.1111/jocn.17017 |
|  | Alcohol Use (current/past/never) | Silveira, E. A., Vinícius-Souza, G., Pereira, C. C., de Oliveira, C., Noll, M., & Pagotto, V. (2023). Osteosarcopenia later in life: prevalence and associated risk factors. Clinical Nutrition ESPEN, 58, 213-220.  <https://doi.org/10.1016/j.clnesp.2023.08.030>  Wang, G., Li, D. Y., Vance, D. E., & Li, W. (2023). Alcohol Use Disorder as a Risk Factor for Cognitive Impairment. Journal of Alzheimer's disease : JAD, 94(3), 899–907. <https://doi.org/10.3233/JAD-230181>  Wei, X., Chen, Y., Qin, J., Yang, Y., Yang, T., Yan, F., Zhang, Z., Han, L., & Ma, Y. (2024). Factors associated with the intrinsic capacity in older adults: A scoping review. Journal of clinical nursing, 33(5), 1739–1750. https://doi.org/10.1111/jocn.17017 |
|  | Physical Activity (low/moderate/high) | Inoue, T., Maeda, K., Nagano, A., Shimizu, A., Ueshima, J., Murotani, K., ... & Tsubaki, A. (2021). Related factors and clinical outcomes of osteosarcopenia: a narrative review. Nutrients, 13(2), 291.  <https://doi.org/10.3390/nu13020291>  Iso-Markku P, Aaltonen S, Kujala UM, et al. (2024). Physical Activity and Cognitive Decline Among Older Adults: A Systematic Review and Meta-Analysis. JAMA Netw Open. 7(2):e2354285. doi:10.1001/jamanetworkopen.2023.54285  Ma L. (2025). Physical activity, sedentary behaviour, and intrinsic capacity at older ages: get active!. The lancet. Healthy longevity, 6(5), 100687. https://doi.org/10.1016/j.lanhl.2025.100687 |
|  | Polypharmacy (yes/no) | Tan, Y.Q., Ng, D.X., Gunasekaran, K. et al. Clinical characteristics and risk factors of osteoporosis among older Asian men with type-2 diabetes mellitus, hypertension, or hyperlipidaemia. (2024). Arch Osteoporos 19, 83. <https://doi.org/10.1007/s11657-024-01442-y>  Yu, X., Qian, Y., Zhang, Y., Chen, Y., & Wang, M. (2024). Association between polypharmacy and cognitive impairment in older adults: A systematic review and meta-analysis. Geriatric nursing (New York, N.Y.), 59, 330–337. <https://doi.org/10.1016/j.gerinurse.2024.07.005>  Meng, L. C., Hsiao, F. Y., Huang, S. T., Lu, W. H., Peng, L. N., & Chen, L. K. (2022). Intrinsic Capacity Impairment Patterns and their Associations with Unfavorable Medication Utilization: A Nationwide Population-Based Study of 37,993 Community-Dwelling Older Adults. The journal of nutrition, health & aging, 26(10), 918–925. https://doi.org/10.1007/s12603-022-1847-z |
|  | Glucocorticoid use (yes/no) | Hirschfeld, H. P., Kinsella, R., & Duque, G. J. O. I. (2017). Osteosarcopenia: where bone, muscle, and fat collide. Osteoporosis international, 28(10), 2781-2790.  <https://doi.org/10.1007/s00198-017-4151-8>  De Alcubierre, D., Ferrari, D., Mauro, G., Isidori, A. M., Tomlinson, J. W., & Pofi, R. (2023). Glucocorticoids and cognitive function: a walkthrough in endogenous and exogenous alterations. Journal of endocrinological investigation, 46(10), 1961–1982. https://doi.org/10.1007/s40618-023-02091-7 |
|  | Calcium/Vitamin D supplementation (yes/no) | Silveira, E. A., Vinícius-Souza, G., Pereira, C. C., de Oliveira, C., Noll, M., & Pagotto, V. (2023). Osteosarcopenia later in life: prevalence and associated risk factors. Clinical Nutrition ESPEN, 58, 213-220.  <https://doi.org/10.1016/j.clnesp.2023.08.030>  Tay, L., Tay, E. L., Mah, S. M., Latib, A., & Ng, Y. S. (2022). Intrinsic capacity rather than intervention exposure influences reversal to robustness among prefrail community-dwelling older adults: A non-randomized controlled study of a multidomain exercise and nutrition intervention. Frontiers in medicine, 9, 971497. https://doi.org/10.3389/fmed.2022.971497 |
|  | Heart rate medication (yes/no) | Fahimfar, N., Yousefi, S., Noorali, S. et al. (2022). The association of cardio-metabolic risk factors and history of falling in men with osteosarcopenia: a cross-sectional analysis of Bushehr Elderly Health (BEH) program. BMC Geriatr 22, 46. https://doi.org/10.1186/s12877-021-02657-1  Zuo, W., & Wu, J. (2022). The interaction and pathogenesis between cognitive impairment and common cardiovascular diseases in the elderly. Therapeutic advances in chronic disease, 13, 20406223211063020. <https://doi.org/10.1177/20406223211063020>  Wei, X., Chen, Y., Qin, J., Yang, Y., Yang, T., Yan, F., Zhang, Z., Han, L., & Ma, Y. (2024). Factors associated with the intrinsic capacity in older adults: A scoping review. Journal of clinical nursing, 33(5), 1739–1750. https://doi.org/10.1111/jocn.17017 |
|  | Mood Medication (Yes/No) | Gebara, M. A., Shea, M. L., Lipsey, K. L., Teitelbaum, S. L., Civitelli, R., Müller, D. J., Reynolds, C. F., 3rd, Mulsant, B. H., & Lenze, E. J. (2014). Depression, antidepressants, and bone health in older adults: a systematic review. Journal of the American Geriatrics Society, 62(8), 1434–1441. <https://doi.org/10.1111/jgs.12945>  Wu, J., Tang, J., Huang, D., Wang, Y., Zhou, E., Ru, Q., Xu, G., Chen, L., & Wu, Y. (2025). Study on the comorbid mechanisms of sarcopenia and late-life depression. Behavioural Brain Research, 485, 1–25. <https://doi.org/10.1016/j.bbr.2025.115538>  Saczynski, J. S., Rosen, A. B., McCammon, R. J., Zivin, K., Andrade, S. E., Langa, K. M., Vijan, S., Pirraglia, P. A., & Briesacher, B. A. (2015). Antidepressant Use and Cognitive Decline: The Health and Retirement Study. The American journal of medicine, 128(7), 739–746. https://doi.org/10.1016/j.amjmed.2015.01.007 |
|  | Self-reported Diabetes (yes/no) | Tan, Y.Q., Ng, D.X., Gunasekaran, K. et al. Clinical characteristics and risk factors of osteoporosis among older Asian men with type-2 diabetes mellitus, hypertension, or hyperlipidaemia. (2024). Arch Osteoporos 19, 83. <https://doi.org/10.1007/s11657-024-01442-y>  Sebastian, M. J., Khan, S. K., Pappachan, J. M., & Jeeyavudeen, M. S. (2023). Diabetes and cognitive function: An evidence-based current perspective. World journal of diabetes, 14(2), 92–109. <https://doi.org/10.4239/wjd.v14.i2.92>  Wei, X., Chen, Y., Qin, J., Yang, Y., Yang, T., Yan, F., Zhang, Z., Han, L., & Ma, Y. (2024). Factors associated with the intrinsic capacity in older adults: A scoping review. Journal of clinical nursing, 33(5), 1739–1750. https://doi.org/10.1111/jocn.17017 |
|  | Self-reported Hyperlipidaemia (yes/no) | Jiang, Y., Xu, B., Zhang, K. et al. (2023). The association of lipid metabolism and sarcopenia among older patients: a cross-sectional study. Sci Rep 13, 17538. <https://doi.org/10.1038/s41598-023-44704-4>  Hu, N., Yan, Y., Wei, S., Lv, H., Dang, L., Gao, L., Wang, J., Guo, X., Wang, J., Qu, Q., & Shang, S. (2025). The relationship between trajectories of serum lipids and cognitive impairment in middle aged and elderly: A 6-year prospective cohort study in Xi'an, China. Medicina clinica, 165(2), 106959. <https://doi.org/10.1016/j.medcli.2025.106959>  Wei, X., Chen, Y., Qin, J., Yang, Y., Yang, T., Yan, F., Zhang, Z., Han, L., & Ma, Y. (2024). Factors associated with the intrinsic capacity in older adults: A scoping review. Journal of clinical nursing, 33(5), 1739–1750. https://doi.org/10.1111/jocn.17017 |
|  | Self-reported Hypertension (yes/no) | Fahimfar, N., Yousefi, S., Noorali, S. et al. (2022). The association of cardio-metabolic risk factors and history of falling in men with osteosarcopenia: a cross-sectional analysis of Bushehr Elderly Health (BEH) program. BMC Geriatr 22, 46. https://doi.org/10.1186/s12877-021-02657-1  Canavan, M., & O'Donnell, M. J. (2022). Hypertension and Cognitive Impairment: A Review of Mechanisms and Key Concepts. Frontiers in neurology, 13, 821135. <https://doi.org/10.3389/fneur.2022.821135>  Wei, X., Chen, Y., Qin, J., Yang, Y., Yang, T., Yan, F., Zhang, Z., Han, L., & Ma, Y. (2024). Factors associated with the intrinsic capacity in older adults: A scoping review. Journal of clinical nursing, 33(5), 1739–1750. https://doi.org/10.1111/jocn.17017 |
|  | Frailty (non-frailty, pre- or intermediate frail, frailty) | Fatima, M., Kirk, B., Vogrin, S., Lee, W. J., Peng, L. N., Chen, L. K., & Duque, G. (2025). Osteosarcopenia and frailty risk in community-dwelling older adults: A follow-up of the I-Lan Longitudinal Aging Study. Archives of gerontology and geriatrics, 136, 105888. https://doi.org/10.1016/j.archger.2025.105888  Brigola, A. G., Rossetti, E. S., Dos Santos, B. R., Neri, A. L., Zazzetta, M. S., Inouye, K., & Pavarini, S. C. I. (2015). Relationship between cognition and frailty in elderly: A systematic review. Dementia & neuropsychologia, 9(2), 110–119. https://doi.org/10.1590/1980-57642015DN92000005 |

**Supplementary Table 6: Additional characteristics of the study sample at baseline**

| **Characteristic** | **Total**  **(N = 2,291) *** | **No Osteosarcopenia (N = 1,127)** | **Possible Osteosarcopenia (N= 1,153)** | **p-value** |
| --- | --- | --- | --- | --- |
| **Current smoker (N, %) **** |  |  |  | 0.891 |
| No | 2,117 (92.4) | 1,040(92.3) | 1,067(92.5) |  |
| Yes | 173 (7.6) | 87(7.7) | 85(7.4) |  |
| **Alcohol consumption status (N, %) **** |  |  |  | 0.003 |
| Currently consume | 293 (12.8) | 176(15.6) | 116(10.1) |  |
| Consumed in the past | 193 (8.4) | 84(7.5) | 109(9.5) |  |
| Never consumed | 1,804 (78.7) | 867(76.9) | 927(80.4) |  |
| **Polypharmacy (N, %)** |  |  |  | <0.001 |
| No | 1,717 (75.00) | 906(80.4) | 803(69.6) |  |
| Yes | 574 (25.1) | 221(19.6) | 350(30.4) |  |
| **Taking glucocorticoids (N, %) **** |  |  |  | 0.972 |
| No | 2,270 (99.1) | 1,117(99.1) | 1,142(99.1) |  |
| Yes | 14 (0.6) | 6(0.5) | 8(0.7) |  |
| **Taking calcium or Vitamin D supplementation (N, %) **** |  |  |  | 0.202 |
| No | 1,274 (55.6) | 643(57.1) | 623(54.0) |  |
| Yes | 1,005 (43.9) | 481(42.7) | 521(45.2) |  |
| **Taking medication to improve mood (N, %) **** |  |  |  | 0.884 |
| No | 2,257 (98.5) | 1,111(98.6) | 1,135(98.4) |  |
| Yes | 33 (1.4) | 16(1.4) | 17(1.5) |  |
| **Taking medication for heart rate (N, %)** |  |  |  | <0.001 |
| No | 2,062 (90.0) | 1,047(92.9) | 1,005(87.2) |  |
| Yes | 229 (10.0) | 80(7.1) | 148(12.8) |  |
| **Self-reported diabetes status (N, %)** |  |  |  | 0.053 |
| No | 1,593 (69.5) | 810(71.9) | 775(67.2) |  |
| Yes | 698 (30.5) | 317(28.1) | 378(32.8) |  |
| **Self-reported hyperlipidaemia status (N, %)** |  |  |  | 0.150 |
| No | 903 (39.4) | 455(40.4) | 441(38.3) |  |
| Yes | 1,388 (60.6) | 672(59.6) | 712(61.8) |  |
| **Self-reported hypertension status (N, %)** |  |  |  | 0.005 |
| No | 904 (39.5) | 476(42.2) | 421(36.5) |  |
| Yes | 1,387 (60.5) | 651(57.8) | 732(63.5) |  |
| **Physical activity levels (N, %) **** |  |  |  | <0.001 |
| Low level | 749 (32.7) | 314(27.9) | 429(37.2) |  |
| Moderate level | 1,134 (49.5) | 576(51.1) | 553(48.0) |  |
| High level | 389 (17.0) | 226(20.1) | 163(14.1) |  |
| **Resilience Scale (Mean, SD)** | 3.8 (0.45) | 3.8 (0.5) | 3.8 (0.5) | 0.05 |
| **Number of chronic conditions (N, %)** |  |  |  | <0.001 |
| 0 chronic conditions | 302 (13.2) | 162(144) | 138(12.0) |  |
| 1-3 chronic conditions | 1398 (61.) | 727(64.5) | 663(58.0) |  |
| ≥ 4 chronic conditions | 591 (25.8) | 238(21.1) | 352(30.5) |  |
| **IADL (Mean, SD)** | 14.7 (2.6) | 15.5 (1.6) | 14.0 (3.2) | <0.001 |
| **EQ_5D Quality of Life (Mean, SD)** | 0.9 (0.2) | 0.9 (0.1) | 0.9 (0.2) | <0.001 |
| **Number of medications (Mean, SD)** | 3.8 (2.6) | 3.4 (2.4) | 4.1 (2.7) | <0.001 |
| **ICON-Falls Efficacy Scale (Fear of Falling) (Mean, SD)** | 17.0 (6.9) | 15.2 (5.8) | 18.8 (7.5) | <0.001 |
| **Lubben social network scale (Mean, SD)** | 13.8 (5.7) | 14.5 (5.5) | 13.0 (5.7) | <0.001 |
| **Loneliness score (Mean, SD)** | 3.6 (1.3) | 3.6 (1.2) | 3.7 (1.3) | <0.001 |
| **Number of insomnia symptoms (Mean, SD)** | 1.7 (1.6) | 1.6 (1.5) | 1.7 (1.6) | 0.006 |
| **Frailty (N, %) **** |  |  |  | <0.001 |
| Non-frail | 144 (6.3) | 144 (12.8) | 0 (0.0) |  |
| Pre- or intermediate frail | 1525 (66.6) | 771 (68.4) | 754 (65.4) |  |
| Frailty | 390 (17.0) | 118 (10.5) | 272 (23.6) |  |

* 11 participants were unable to determine osteo-sarcopenia status, hence total number cannot add up to 2,291.

** Contains a missing value, thus percentages might not add up to 100%.

IADL: Independent activities of daily living

**Supplementary Table 7: Association between possible osteosarcopenia and intact locomotion capacity of IC at baseline (n = 2,280*)**

|  | **Locomotion capacity (Intact)** | | |
| --- | --- | --- | --- |
|  | **Odd Ratio** | **95% CI** | **p-value** |
| **Osteosarcopenia (Ref: No possible osteosarcopenia)** |  |  |  |
| Possible osteosarcopenia | 0.80 | (0.52, 1.22) | 0.297 |
| **Age** | 0.90 | (0.87, 0.93) | <0.001 |
| **Gender (Ref: Male)** |  |  |  |
| Female | 0.68 | (0.47, 0.98) | 0.036 |
| **Financial resources (Ref: More than adequate)** |  |  |  |
| Adequate | 0.50 | (0.21, 1.19) | 0.118 |
| Occasionally adequate | 0.38 | (0.14, 1.02) | 0.055 |
| Usually inadequate | 0.26 | (0.07, 1.05) | 0.059 |
| **Alcohol consumption (Ref: Currently consume)** |  |  |  |
| Consumed in the past but not currently | 0.61 | (0.28, 1.30) | 0.197 |
| Never consumed | 0.59 | (0.36, 0.96) | 0.034 |
| **Physical activity level (Ref: Low level)** |  |  |  |
| Moderate level | 1.13 | (0.74, 1.72) | 0.580 |
| High level | 1.21 | (0.72, 2.05) | 0.471 |

CI: confidence interval

* Osteosarcopenia status could not be determined for 11 participants, thus they were excluded for the analysis.

**Supplementary Table 8: Association between possible osteosarcopenia and intact sensory capacity of IC at baseline (n = 2,280*)**

|  | **Sensory capacity (Intact)** | | |
| --- | --- | --- | --- |
|  | **Odd Ratio** | **95% CI** | **p-value** |
| **Osteosarcopenia (Ref: No possible osteosarcopenia)** |  |  |  |
| Possible osteo-sarcopenia | 0.77 | (0.59, 1.02) | 0.067 |
| **Age** | 0.95 | (0.93, 0.97) | <0.001 |
| **Gender (Ref: Male)** |  |  |  |
| Female | 1.45 | (1.13, 1.85) | 0.003 |
| **Ethnic (Ref: Chinese)** |  |  |  |
| Malay | 1.17 | (0.84, 1.63) | 0.356 |
| Indian | 1.71 | (1.15, 2.55) | 0.008 |
| Other | 2.44 | (1.00, 5.97) | 0.051 |
| **Financial resources (Ref: More than adequate)** |  |  |  |
| Adequate | 0.99 | (0.49, 1.98) | 0.976 |
| Occasionally adequate | 0.88 | (0.42, 1.85) | 0.745 |
| Usually inadequate | 0.29 | (0.12, 0.71) | 0.007 |
| **Number of chronic conditions (Ref: No)** |  |  |  |
| 1-3 chronic conditions | 0.96 | (0.66, 1.41) | 0.849 |
| ≥ 4 chronic conditions | 0.41 | (0.27, 0.63) | <0.001 |
| **Alcohol consumption (Ref: Currently consume)** |  |  |  |
| Consumed in the past but not currently | 1.96 | (1.14, 3.36) | 0.015 |
| Never consumed | 1.19 | (0.82, 1.74) | 0.355 |
| **ICON-Falls Efficacy Scale (Fear of Falling)** | 0.95 | (0.93, 0.97) | <0.001 |
| **Loneliness score** | 0.86 | (0.79, 0.95) | 0.002 |

CI: confidence interval

* Osteosarcopenia status could not be determined for 11 participants, thus they were excluded for the analysis.

**Supplementary Table 9: Association between possible osteosarcopenia and intact psychological capacity of IC at baseline (n = 2,280*)**

|  | **Psychological capacity (Intact)** | | |
| --- | --- | --- | --- |
|  | **Odd Ratio** | **95% CI** | **p-value** |
| **Osteosarcopenia (Ref: No possible osteosarcopenia)** |  |  |  |
| Possible osteosarcopenia | 0.92 | (0.54, 1.56) | 0.759 |
| **Age** | 1.00 | (0.97, 1.04) | 0.792 |
| **Ethnic (Ref: Chinese)** |  |  |  |
| Malay | 1.54 | (0.80, 2.99) | 0.199 |
| Indian | 0.76 | (0.42, 1.35) | 0.345 |
| Other | 0.77 | (0.23, 2.57) | 0.677 |
| **Household type** |  |  |  |
| HDB flat (3-4 rooms) | 0.85 | (0.44, 1.68) | 0.647 |
| (5 room and above) and Private house | 1.88 | (0.83, 4.25) | 0.129 |
| **Frailty (Ref: Non-frail)** |  |  |  |
| Pre- or intermediate frail | 0.33 | (0.04, 2.54) | 0.285 |
| Frailty | 0.10 | (0.01, 0.82) | 0.032 |
| **Loneliness score** | 0.70 | (0.59, 0.82) | <0.001 |
| **Number of medications** | 0.88 | (0.81, 0.95) | 0.001 |
| **Resilience scale** | 3.69 | (2.44, 5.57) | <0.001 |

CI: confidence interval

HDB: Housing & Development Board

* Osteosarcopenia status could not be determined for 11 participants, thus they were excluded for the analysis.

**Supplementary Table 10: Association between possible osteosarcopenia and intact vitality capacity of IC at baseline (n = 2,280*)**

|  | **Vitality capacity (Intact)** | | |
| --- | --- | --- | --- |
|  | **Odd ratio** | **95% CI** | **p-value** |
| **Osteosarcopenia (Ref: No possible osteosarcopenia)** |  |  |  |
| Possible osteosarcopenia | 0.32 | (0.20, 0.52) | <0.001 |
| **Financial resources (Ref: More than adequate)** |  |  |  |
| Adequate | 1.20 | (0.41, 3.54) | 0.737 |
| Occasionally adequate | 0.63 | (0.21, 1.91) | 0.414 |
| Usually inadequate | 1.21 | (0.27, 5.38) | 0.803 |
| **Living arrangement (Ref: Living alone)** |  |  |  |
| Living with Child and Spouse | 2.31 | (1.24, 4.30) | 0.008 |
| Living with Child Only | 1.42 | (0.76, 2.64) | 0.270 |
| Living with Spouse Only | 1.76 | (0.99, 3.13) | 0.054 |
| Living with Others Only (Include Foreign Domestic Worker) | 1.54 | (0.78, 3.04) | 0.218 |
| **Vitamin D/Calcium intake (Ref: No)** |  |  |  |
| Yes | 1.01 | (0.69, 1.48) | 0.954 |
| **Using medication to improve mood (Ref: No)** |  |  |  |
| Yes | 0.41 | (0.13, 1.27) | 0.122 |
| **EQ_5D Quality of life** | 1.17 | (0.31, 4.45) | 0.813 |
| **Frailty (Ref: Non-frail)** |  |  |  |
| Pre- or intermediate frail | 1.80 | (0.71, 4.57) | 0.215 |
| Frailty | 1.00 | (0.37, 2.72) | 0.992 |

CI: confidence interval

HDB: Housing & Development Board

* Osteosarcopenia status could not be determined for 11 participants, thus they were excluded for the analysis.

**Supplementary Table 11: Association between possible osteosarcopenia and intact capacity in the cognition domain of intrinsic capacity at baseline (n=2,280*)**

|  | **Cognition capacity (Intact)** | | |
| --- | --- | --- | --- |
|  | **Odd Ratio** | **95% CI** | **p-value** |
| **Osteosarcopenia (Ref: No possible osteosarcopenia)** |  |  |  |
| Possible osteosarcopenia | 0.45 | (0.34, 0.60) | <0.001 |
| **Age** | 0.88 | (0.86, 0.91) | <0.001 |
| **Ethnicity (Ref: Chinese)** |  |  |  |
| Malay | 0.34 | (0.23, 0.49) | <0.001 |
| Indian | 0.50 | (0.33, 0.76) | 0.001 |
| Other | 0.53 | (0.21, 1.38) | 0.196 |
| **Financial resources (Ref: More than adequate)** |  |  |  |
| Adequate | 0.53 | (0.26, 1.08) | 0.079 |
| Occasionally adequate | 0.15 | (0.07, 0.33) | <0.001 |
| Usually inadequate | 0.20 | (0.07, 0.57) | 0.002 |
| **Instrumental activities of daily living** | 1.28 | (1.18, 1.39) | <0.001 |
| **Loneliness score** | 0.94 | (0.85, 1.05) | 0.274 |
| **Lubben Social Network Scale** | 1.07 | (1.05, 1.10) | <0.001 |

CI: confidence interval
* Osteosarcopenia status could not be determined for 11 participants, who were excluded for this analysis

**Supplementary Table 12: Predicted mean mini-MoCA scores and marginal effects of osteosarcopenia by baseline cognition level**

| **Baseline cognitive capacity** | **Possible osteosarcopenia status** | **Predicted mean of mini-MoCA score** | **Marginal effects** |
| --- | --- | --- | --- |
| Loss | No possible osteosarcopenia | 11.64 (11.34 – 11.94) | Reference |
|  | Possible osteosarcopenia | 11.08 (10.79 – 11.36) | –0.56  (–0.99, –0.13),  p = 0.010 |
| Intact | No possible osteosarcopenia | 12.84 (12.65 – 13.03) | Reference |
|  | Possible osteosarcopenia | 12.82 (12.53 – 13.10) | –0.02 (–0.35, 0.31), p = 0.904 |

**Supplementary Table 13:** **Association between possible osteosarcopenia and delayed recall domain of mini-MoCA at 6-month follow-up (n = 2,047^)**

| **Delay recall domain** | **Coefficient** | **95% CI** | **p-value** |
| --- | --- | --- | --- |
| **Osteo-sarcopenia (Ref: No possible osteo-sarcopenia)** |  |  |  |
| Possible osteo-sarcopenia | -0.01 | (-0.06, 0.05) | 0.800 |
| **Baseline cognitive domain (Ref: Cognitive intact**^¶^**)** |  |  |  |
| Loss in cognitive capacity^¶^ | -0.57 | (-0.61, -0.53) | <0.001 |
| **Osteosarcopenia#Baseline cognitive domain interaction (Ref: No osteosarcopenia# Cognitive intact^$^)** |  |  |  |
| Possible osteosarcopenia#Loss in cognitive capacity ^¶^ | -0.41 | (-0.47, -0.35) | <0.001 |
| **Age** | -0.05 | (-0.06, -0.05) | <0.001 |
| **Gender (Ref: Male)** |  |  |  |
| Female | 0.29 | (0.26, 0.31) | <0.001 |
| **Financial resources (Ref: More than adequate)** |  |  |  |
| Adequate | -0.40 | (-0.49, -0.31) | <0.001 |
| Occasionally adequate | -0.80 | (-0.90, -0.71) | <0.001 |
| Usually inadequate | -0.24 | (-0.35, -0.12) | <0.001 |
| **Household type** |  |  |  |
| HDB flat (3-4 rooms) | -0.16 | (-0.20, -0.12) | <0.001 |
| (5 room and above) and Private house | 0.20 | (0.16, 0.25) | <0.001 |
| **Living arrangement (Ref: Living alone)** |  |  |  |
| Living with Child and Spouse | -0.05 | (-0.10, -0.06) | 0.029 |
| Living with Child Only | -0.18 | (-0.23, -0.13) | <0.001 |
| Living with Spouse Only | 0.13 | (0.08, 0.18) | <0.001 |
| Living with Others Only (Include Foreign Domestic Worker) | -0.01 | (-0.07, 0.04) | 0.670 |
| **Frailty (Ref: Non-frail)** |  |  |  |
| Pre- or intermediate frail | 0.08 | (0.16, 0.14) | 0.013 |
| Frailty | -0.28 | (-0.34, -0.21) | <0.001 |
| **Lubben Social Network Scale** | 0.02 | (0.01, 0.02) | <0.001 |

^: Sample size includes 819 imputed cases

CI: confidence interval

HDB: Housing & Development Board

^¶^ Cognitive impairment: MoCA score ≤ 25; Intact cognitive: MoCA score ≥ 26.

**Supplementary Table 14:** **Association between possible osteosarcopenia and language fluency domain of mini-MoCA at 6-month follow-up (n = 2,047^)**

| **Language fluency domain** | **Coefficient** | **95% CI** | **p-value** |
| --- | --- | --- | --- |
| **Osteosarcopenia (Ref: No possible osteosarcopenia)** |  |  |  |
| Possible osteosarcopenia | -0.14 | (-0.39, 0.10) | 0.242 |
| **Baseline cognitive domain (Ref: Cognitive intact**^¶^**)** |  |  |  |
| Loss in cognitive capacity^¶^ | -0.44 | (-0.55, -0.34) | <0.001 |
| **Osteosarcopenia#Baseline cognitive domain interaction (Ref: No osteosarcopenia# Cognitive intact^$^)** |  |  |  |
| Possible osteosarcopenia#Loss in cognitive capacity ^¶^ | 0.09 | (-0.15, 0.33) | 0.475 |
| **Age** | -0.01 | (-0.02, -0.01) | <0.001 |
| **Ethnic (Ref: Chinese)** |  |  |  |
| Malay | 0.33 | (0.27, 0.38) | <0.001 |
| Indian | 0.26 | (0.21, 0.31) | <0.001 |
| Other | 0.15 | (0.08, 0.21) | <0.001 |
| **Highest educational level (Ref: No formal education)** |  |  |  |
| Primary | 0.19 | (0.14, 0.24) | <0.001 |
| Secondary | 0.41 | (0.35, 0.47) | <0.001 |
| Vocational or Institute of Technical Education (ITE) | 0.21 | (0.01, 0.41) | <0.001 |
| Junior College (JC) or GCE ‘A’ Level | 0.64 | (0.56, 0.71) | <0.001 |
| Polytechnic | 0.53 | (0.44, 0.61) | <0.001 |
| University and above | 0.79 | (0.71, 0.87) | <0.001 |
| **Financial resources (Ref: More than adequate)** |  |  |  |
| Adequate | -0.18 | (-0.26, -0.10) | <0.001 |
| Occasionally adequate | -0.38 | (-0.47, -0.29) | <0.001 |
| Usually inadequate | -0.08 | (-0.19, 0.02) | 0.119 |
| **Alcohol consumption (Ref: Currently consume)** |  |  |  |
| Consumed in the past but not currently | -0.08 | (-0.28, 0.11) | 0.408 |
| Never consumed | 0.01 | (-0.23, 0.20) | 0.883 |
| **Frailty (Ref: Non-frail)** |  |  |  |
| Pre- or intermediate frail | -0.09 | (-0.15, -0.03) | 0.006 |
| Frailty | -0.39 | (-0.54, -0.25) | <0.001 |

^: Sample size includes 799 imputed cases

CI: confidence interval

^¶^ Cognitive impairment: MoCA score ≤ 25; Intact cognitive: MoCA score ≥ 26.

**Supplementary Table 15:** **Association between possible osteosarcopenia and orientation domain of mini-MoCA at 6-month follow-up (n = 2,047^)**

| **Orientation domain** | **Coefficient** | **95% CI** | **p-value** |
| --- | --- | --- | --- |
| **Osteo-sarcopenia (Ref: No osteo-sarcopenia)** |  |  |  |
| Possible osteo-sarcopenia | -0.03 | (-0.05, 0.01) | 0.031 |
| **Baseline cognitive domain (Ref: Cognitive intact**^¶^**)** |  |  |  |
| Loss in cognitive capacity^¶^ | -0.12 | (-0.15, -0.09) | <0.001 |
| **Osteosarcopenia#Baseline cognitive domain interaction (Ref: No osteosarcopenia# Cognitive intact^$^)** |  |  |  |
| Possible osteosarcopenia#Loss in cognitive capacity ^¶^ | -0.12 | (-0.15, -0.09) | <0.001 |
| **Age** | -0.01 | (-0.01, -0.01) | <0.001 |
| **Financial resources (Ref: More than adequate)** |  |  |  |
| Adequate | -0.08 | (-0.11, 0.05) | <0.001 |
| Occasionally adequate | -0.10 | (-0.14, -0.07) | <0.001 |
| Usually inadequate | 0.08 | (0.04, 0.11) | <0.001 |
| **Living arrangement (Ref: Living alone)** |  |  |  |
| Living with Child and Spouse | -0.01 | (-0.02, 0.02) | 0.852 |
| Living with Child Only | -0.08 | (-0.10, -0.06) | <0.001 |
| Living with Spouse Only | 0.04 | (-0.02, 0.02) | 0.710 |
| Living with Others Only (Include Foreign Domestic Worker) | -0.19 | (-0.23, -0.16) | <0.009 |
| **Number of chronic conditions (Ref: No)** |  |  |  |
| 1-3 chronic conditions | 0.02 | (-0.01, 0.03) | 0.069 |
| ≥ 4 chronic conditions | 0.15 | (0.13, 0.17) | <0.001 |
| **Instrumental activities of daily living** | 0.06 | (0.06, 0.06) | <0.001 |

^: Sample size includes 813 imputed cases

CI: confidence interval

^¶^ Cognitive impairment: MoCA score ≤ 25; Intact cognitive: MoCA score ≥ 26.

**Supplementary Table 16: Association between possible osteosarcopenia and cognitive function (measured by the mini-MoCA) as well as each domain at the 6-month follow-up among non-imputed cases (n=1,228)**

|  | **Coefficient** | **95% CI** | **p-value** |
| --- | --- | --- | --- |
| ***Total cognition function*** |  |  |  |
| **Osteosarcopenia (Ref: No possible osteosarcopenia)** |  |  |  |
| Possible osteosarcopenia | -0.02 | (-0.35, 0.31) | 0.904 |
| **Baseline cognition capacity (Ref: Intact cognitive capacity**^¶^**)** |  |  |  |
| Loss in cognitive capacity^¶^ | -1.20 | (-1.52, -0.87) | <0.001 |
| **Osteosarcopenia#Baseline cognition capacity interaction (Ref: No possible osteosarcopenia#Intact cognitive capacity^$^)** |  |  |  |
| Possible osteosarcopenia#Loss in cognitive capacity ^¶^ | -0.54 | (-1.06, -0.03) | 0.038 |
| **Age** | -0.08 | (-0.10, -0.06) | <0.001 |
| **Financial resources (Ref: More than adequate)** |  |  |  |
| Adequate | -0.86 | (-1.33, -0.39) | <0.001 |
| Occasionally adequate | -1.48 | (-2.05, -0.91) | <0.001 |
| Usually inadequate | -0.61 | (-1.51, 0.28) | 0.177 |
| **Number of chronic conditions (Ref: No)** |  |  |  |
| 1-3 chronic conditions | 0.15 | (-0.23, 0.53) | 0.440 |
| ≥ 4 chronic conditions | 0.47 | (0.03, 0.91) | 0.037 |
| **Frailty (Ref: Non-frail)** |  |  |  |
| Pre- or intermediate frail | 0.07 | (-0.34, 0.48) | 0.730 |
| Frailty | -0.68 | (-1.23, -0.14) | 0.014 |
| **Instrumental activities of daily living** | 0.10 | (-0.01, 0.21) | 0.086 |
| **Lubben Social Network Scale** | 0.04 | (0.02, 0.07) | <0.001 |
| ***Language fluency domain (N = 1,248)*** |  |  |  |
| **Osteosarcopenia (Ref: No osteosarcopenia)** |  |  |  |
| Possible osteosarcopenia* | -0.01 | (-0.16, 0.14) | 0.862 |
| ***Orientation domain (N = 1,234)*** |  |  |  |
| **Osteosarcopenia (Ref: No osteosarcopenia)** |  |  |  |
| Possible osteosarcopenia** | -0.01 | (-0.10, 0.08) | 0.823 |
| ***Delayed recall domain (N = 1,228)*** |  |  |  |
| **Osteosarcopenia (Ref: No osteosarcopenia)** |  |  |  |
| Possible osteosarcopenia*** | -0.20 | (-0.39, -0.01) | 0.042 |
| **Osteosarcopenia#Baseline cognitive domain interaction (Ref: No osteosarcopenia# Cognitive intact^$^)** |  |  |  |
| Possible osteosarcopenia#Loss in cognitive capacity ^¶^ | -0.40 | (-0.74, -0.06) | 0.023 |

CI: confidence interval

^¶^ Loss in cognitive capacity: MoCA score ≤ 25; Intact cognitive capacity: MoCA score ≥ 26.

* Adjusted for Age, Ethnicity, Highest educational level, Financial resources, Alcohol consumption, Frailty, Baseline cognitive capacity.

** Adjusted for Age, Financial resources, Living arrangement, Number of chronic conditions, Instrumental activities of daily living, Baseline cognitive capacity.

*** Adjusted for Age, Gender, Financial resources, Household type, Living arrangement, Frailty, Lubben Social Network, Baseline cognitive capacity.

**Supplementary Table 17:** **Association between possible osteosarcopenia and delayed recall domain of mini-MoCA at 6-month follow-up among non-imputed cases (n = 1,228)**

| **Delay recall domain** | **Coefficient** | **95% CI** | **p-value** |
| --- | --- | --- | --- |
| **Osteo-sarcopenia (Ref: No possible osteo-sarcopenia)** |  |  |  |
| Possible osteo-sarcopenia | -0.20 | (-0.39, -0.01) | 0.042 |
| **Baseline cognitive domain (Ref: Cognitive intact**^¶^**)** |  |  |  |
| Loss in cognitive capacity^¶^ | -0.56 | (-0.79, -0.33) | <0.001 |
| **Osteosarcopenia#Baseline cognitive domain interaction (Ref: No osteosarcopenia# Cognitive intact^$^)** |  |  |  |
| Possible osteosarcopenia#Loss in cognitive capacity ^¶^ | -0.40 | (-0.74, -0.06) | 0.023 |
| **Age** | -0.05 | (-0.07, -0.04) | <0.001 |
| **Gender (Ref: Male)** |  |  |  |
| Female | 0.25 | (0.08, 0.42) | 0.005 |
| **Financial resources (Ref: More than adequate)** |  |  |  |
| Adequate | -0.42 | (-0.74, -0.09) | 0.012 |
| Occasionally adequate | -0.80 | (-1.20, -0.40) | <0.001 |
| Usually inadequate | -0.22 | (-0.86, 0.42) | 0.493 |
| **Household type** |  |  |  |
| HDB flat (3-4 rooms) | -0.16 | (-0.45, 0.14) | 0.295 |
| (5 room and above) and Private house | 0.22 | (-0.11, 0.55) | 0.184 |
| **Living arrangement (Ref: Living alone)** |  |  |  |
| Living with Child and Spouse | -0.13 | (-0.44, 0.18) | 0.411 |
| Living with Child Only | -0.23 | (-0.57, 0.12) | 0.201 |
| Living with Spouse Only | 0.08 | (-0.22, 0.37) | 0.607 |
| Living with Others Only (Include Foreign Domestic Worker) | -0.10 | (-0.44, 0.24) | 0.556 |
| **Frailty (Ref: Non-frail)** |  |  |  |
| Pre- or intermediate frail | 0.13 | (-0.16, 0.42) | 0.388 |
| Frailty | -0.26 | (-0.62, 0.11) | 0.170 |
| **Lubben Social Network Scale** | 0.02 | (0.01, 0.04) | 0.010 |

CI: confidence interval

HDB: Housing & Development Board

^¶^ Cognitive impairment: MoCA score ≤ 25; Intact cognitive: MoCA score ≥ 26.

**Supplementary Table 18:** **Association between possible osteosarcopenia and language fluency domain of mini-MoCA at 6-month follow-up among non-imputed cases (n = 1,248)**

| **Language fluency domain** | **Coefficient** | **95% CI** | **p-value** |
| --- | --- | --- | --- |
| **Osteosarcopenia (Ref: No possible osteosarcopenia)** |  |  |  |
| Possible osteosarcopenia | -0.01 | (-0.16, 0.14) | 0.862 |
| **Baseline cognitive domain (Ref: Cognitive intact**^¶^**)** |  |  |  |
| Loss in cognitive capacity^¶^ | -0.39 | (-0.53, -0.25) | <0.001 |
| **Osteosarcopenia#Baseline cognitive domain interaction (Ref: No osteosarcopenia# Cognitive intact^$^)** |  |  |  |
| Possible osteosarcopenia#Loss in cognitive capacity ^¶^ | -0.03 | (-0.24, 0.18) | 0.778 |
| **Age** | -0.02 | (-0.02, -0.01) | <0.001 |
| **Ethnic (Ref: Chinese)** |  |  |  |
| Malay | 0.32 | (0.17, 0.47) | <0.001 |
| Indian | 0.21 | (0.06, 0.35) | 0.004 |
| Other | 0.03 | (-0.26, 0.33) | 0.825 |
| **Highest educational level (Ref: No formal education)** |  |  |  |
| Primary | 0.20 | (0.01, 0.39) | 0.040 |
| Secondary | 0.43 | (0.22, 0.63) | <0.001 |
| Vocational or Institute of Technical Education (ITE) | 0.31 | (-0.11, 0.73) | 0.144 |
| Junior College (JC) or GCE ‘A’ Level | 0.67 | (0.43, 0.91) | <0.001 |
| Polytechnic | 0.56 | (0.28, 0.85) | <0.001 |
| University and above | 0.71 | (0.47, 0.95) | <0.001 |
| **Financial resources (Ref: More than adequate)** |  |  |  |
| Adequate | -0.10 | (-0.30, 0.10) | 0.310 |
| Occasionally adequate | -0.28 | (-0.53, -0.04) | 0.023 |
| Usually inadequate | 0.00 | (-0.34, 0.33) | 0.985 |
| **Alcohol consumption (Ref: Currently consume)** |  |  |  |
| Consumed in the past but not currently | -0.20 | (-0.39, -0.01) | 0.042 |
| Never consumed | -0.16 | (-0.29, -0.03) | 0.019 |
| **Frailty (Ref: Non-frail)** |  |  |  |
| Pre- or intermediate frail | -0.06 | (-0.21, 0.08) | 0.405 |
| Frailty | -0.35 | (-0.56, -0.15) | 0.001 |

CI: confidence interval

^¶^ Cognitive impairment: MoCA score ≤ 25; Intact cognitive: MoCA score ≥ 26.

**Supplementary Table 19:** **Association between possible osteosarcopenia and orientation domain of mini-MoCA at 6-month follow-up among non-imputed cases (n = 1,234)**

| **Orientation domain** | **Coefficient** | **95% CI** | **p-value** |
| --- | --- | --- | --- |
| **Osteo-sarcopenia (Ref: No osteo-sarcopenia)** |  |  |  |
| Possible osteo-sarcopenia | -0.01 | (-0.10, 0.08) | 0.823 |
| **Baseline cognitive domain (Ref: Cognitive intact**^¶^**)** |  |  |  |
| Loss in cognitive capacity^¶^ | -0.13 | (-0.21, -0.04) | 0.005 |
| **Osteosarcopenia#Baseline cognitive domain interaction (Ref: No osteosarcopenia# Cognitive intact^$^)** |  |  |  |
| Possible osteosarcopenia#Loss in cognitive capacity ^¶^ | -0.14 | (-0.28, 0.01) | 0.075 |
| **Age** | -0.01 | (-0.01, 0.00) | 0.036 |
| **Financial resources (Ref: More than adequate)** |  |  |  |
| Adequate | -0.06 | (-0.16, 0.04) | 0.254 |
| Occasionally adequate | -0.10 | (-0.25, 0.04) | 0.150 |
| Usually inadequate | 0.08 | (-0.14, 0.30) | 0.458 |
| **Living arrangement (Ref: Living alone)** |  |  |  |
| Living with Child and Spouse | 0.00 | (-0.11, 0.12) | 0.938 |
| Living with Child Only | -0.05 | (-0.22, 0.12) | 0.551 |
| Living with Spouse Only | 0.03 | (-0.09, 0.14) | 0.648 |
| Living with Others Only (Include Foreign Domestic Worker) | -0.15 | (-0.30, 0.00) | 0.052 |
| **Number of chronic conditions (Ref: No)** |  |  |  |
| 1-3 chronic conditions | 0.01 | (-0.10, 0.12) | 0.802 |
| ≥ 4 chronic conditions | 0.14 | (0.01, 0.27) | 0.029 |
| **Instrumental activities of daily living** | 0.06 | (0.02, 0.10) | 0.001 |

CI: confidence interval

^¶^ Cognitive impairment: MoCA score ≤ 25; Intact cognitive: MoCA score ≥ 26.

**Supplementary Figure 1 Summary of effect modification of baseline osteosarcopenia and cognition status on delayed recall components of Mini-MoCA 6-months post-baseline (n = 2,047).**

**Supplementary Figure 2 Summary of effect modification of baseline osteosarcopenia and cognition status on the orientation component of Mini-MoCA 6-months post-baseline (n = 2,047).**
